# Osmotrophy of dissolved organic compounds by coccolithophore populations: Fixation into particulate organic and inorganic carbon

**DOI:** 10.1126/sciadv.adf6973

**Published:** 2023-05-24

**Authors:** William M. Balch, David T. Drapeau, Nicole Poulton, Stephen D. Archer, Carmen Cartisano, Craig Burnell, Jelena Godrijan

**Affiliations:** ^1^ Bigelow Laboratory for Ocean Sciences, 60 Bigelow Dr., East Boothbay, ME 04544, USA.; ^2^ Division for Marine and Environmental Research, Ruđer Bošković Institute, Zagreb, Croatia.

## Abstract

Coccolithophores are typically thought of as photoautotrophs, yet a few genera inhabit sub-euphotic environments with insufficient light for photosynthesis, suggesting that other carbon acquisition strategies are likely. Field experiments were performed in the northwest Atlantic (a region with potentially abundant coccolithophores). Phytoplankton populations were incubated with ^14^C-labeled dissolved organic carbon (DOC) compounds, acetate, mannitol, and glycerol. Coccolithophores were sorted from these populations 24 hours later using flow cytometry, and DOC uptake was measured. DOC uptake rates were as high as 10^−15^ moles cell^−1^ day^−1^, slow relative to photosynthesis rates (10^−12^ moles cell^−1^ day^−1^). Growth rates on the organic compounds were low, suggesting that osmotrophy plays more of a survival strategy in low-light situations. Assimilated DOC was found in both particulate organic carbon and calcite coccoliths (particulate inorganic carbon), suggesting that osmotrophic uptake of DOC into coccolithophore calcite is a small but notable part of the biological carbon pump and alkalinity pump paradigms.

## Teaser

Coccolithophores in the ocean fix dissolved organic carbon compounds into both organic tissue and inorganic coccoliths.

## INTRODUCTION

### Heterotrophy, mixotrophy, and carbon cycling in marine algae

The reservoir of dissolved organic carbon (DOC) in the world ocean is about 622 Pg, and it consists of a diverse mixture of compounds, >99.9% of which range from being semilabile to ultrarefractory and not biologically available, while <0.1% are considered truly labile and biologically available to microbes (*1*). This labile fraction, however, accounts for 84% of the annual turnover of DOC (*2*), suggesting its importance in the overall carbon cycle. The microalgal utilization of these labile exogenous, reduced, DOC compounds as a metabolic source of carbon and electron donors is deemed “microalgal heterotrophy” (*3*). Mixotrophy is defined as the combination of autotrophy and heterotrophy in the same organism and is frequently found in algae (*4*). Phagotrophy is a subset of heterotrophy in which particles (often whole cells) are engulfed by another cell, encapsulated into a vacuole, after which they are digested. Osmotrophy is another subset of heterotrophy in which DOC is taken up by an organism and used as a source of nutrition.

### Heterotrophy in coccolithophores (Haptophyceae)

Haptophytes are a clade of microalgae exhibiting an extraordinary diversity, and both calcifying and noncalcifying haptophytes dominate global phytoplankton primary production, contributing some 30 to 50% of the total standing stock of photosynthetic algae in the world ocean (*5*). Coccolithophores are calcifying, marine microalgae and are important for ocean seawater chemistry and the global carbon cycle (*6*). It has been known for >80 years that haptophytes can acquire nutrition in a phagotrophic fashion (*7*); however, the implications of this for their physiology, ecology, and biogeochemistry still remain poorly understood. Phagocytic ingestion of particles has been the best-documented aspect of haptophyte mixotrophy (*8*, *9*).

Osmotrophy of dissolved organic matter has remained far-less studied in haptophytes than phagotrophy; evidence has been gathered primarily from culture studies, not field studies. Uptake of certain DOC compounds allows faster recovery of growth from darkness. For example, *Syracosphaera* was shown to use lactate as a carbon source (*10*). Moreover, glycerol allowed *Chrysochromulina kappa* to survive in darkness and recover faster when brought into the light (*10*). Pintner and Provasoli (*11*) observed enhanced survival of axenic cultures of the haptophyte *C. kappa* for 6 months at low light [~0.2 microeinstein (μE) m^−2^ s^−1^; for context, this would be 0.01% of surface summer noontime values in Bermuda] with medium containing 10.8 mM glycerol (a concentration that cells would likely not see in the marine environment). This concentration of glycerol doubled the growth of *C. kappa* under dim light to be as high as cells grown at high light with no glycerol (yet cells could not be grown in complete darkness). At triple this light intensity (0.6 μE m^−2^ s^−1^, still considered “low light”), uptake of several DOC compounds was observed for *Chyrsochromulina* sp. Cultures of *C. strobilus* and *C. brevefilum* lack heterotrophic capability at high light, yet they express the ability at low light when it is most needed (*11*). Osmotrophy of *Chrysochromulina* is thought to allow for growth in low-light polar waters where light is too low for photosynthesis (*12*). There are examples of polar coccolithophores totally lacking chloroplasts (either never acquired or lost secondarily through evolution) that must rely on heterotrophy for survival (*13*).

Coccolithophore osmotrophy even varies with their polymorphic life stages. For example, many coccolithophores have a haplodiplontic life cycle, switching between haploid motile cells covered with holococcoliths (coccoliths made of simple rhombohedral calcite crystals) and a diploid, nonmotile stage covered with heterococcoliths (calcite scales that are more elaborate). Both can divide asexually (*14*). The haploid motile stage of *Coccolithus braarudii* has been observed to augment its growth rate by 40% in the presence of 0.1 mM sodium acetate (*15*). The haploid stages of *C. braarudii* appear to be more mixotrophic (osmotrophic and/or phagotrophic) than the diploid counterparts when grown with soil extract or sodium acetate, while the opposite was observed for cultures of *Calcidiscus leptoporus* grown with soil extract (*15*).

Cultured coccolithophores have been shown to assimilate a wide variety of DOC compounds in the dark via osmotrophy (*16*), and more recently, the kinetics of growth and uptake have been demonstrated for three specific DOC compounds, readily taken up by coccolithophores (and other algae): acetate, mannitol, and glycerol (*17*). While the osmotrophic rates of uptake and growth on these compounds were slow in cultured coccolithophores compared to photosynthetic rates, DOC was rapidly traced into both particulate organic carbon (POC) fractions and particulate inorganic carbon (PIC) fractions.

### Background on the uptake of acetate, mannitol, and glycerol by algae

We review some key studies on the uptake and assimilation of acetate, mannitol, and glycerol by coccolithophores as well as other classes of microalgae to provide context for this work.

### Acetate osmotrophy

A wide variety of microalgae can assimilate acetate. *Chlamydomonas* was first reported to anaerobically photoassimilate acetate (*18*). Two strains of *Chlamydomonas mundana* were later shown to have high growth rates using acetate as a source of carbon during photoheterotrophic metabolism. These strains can also use acetate for the reduction of CO_2_ in the light without net oxygen production, while they carry out normal photosynthesis (*19*). Coral symbiotic zooxanthellae have been shown to take up acetate (*20*), associated with lipogenesis. Moreover, the green microalga, *Chlorella vulgaris*, has been shown to assimilate ^14^C-acetate (*21*), and the activity has been followed into chloroplast lipids (*22*). *Scenedesmus* sp. from the hypolimnion of a pond assimilated acetate as a carbon source in darkness, which also showed enhancement in light (*23*). Other microalgal genera that can assimilate acetate are *Synechococcus*, *Aphanocapsa* (light enhanced) (*24*), and *Chlamydomonas* (light and dark) (*25*). Sloan and Strickland (*26*) first demonstrated minor uptake of ^14^C-acetate by the coccolithophore, *Emiliania huxleyi,* which was only marginally enhanced by light. They also noted that *E. huxleyi* also took up minor amounts of glucose and l-glutamate. Acetate concentration estimates in seawater are rare; they have been measured in coastal pore waters with concentrations of 1 to 12 μM (*27*).

### Mannitol osmotrophy

Mannitol may be one of the most common, naturally occurring sugar alcohols, and it plays multiple roles as carbon source, thermal protectant, osmolyte, compatible solute, and antioxidant (*28*). Naturally occurring picoplankton from the Caribbean and North Atlantic were shown to use radiolabeled mannitol, in light or darkness, even when photosynthesis was inhibited; diatoms were also observed to incorporate mannitol during blooms (*29*). Intracellular concentrations of mannitol paralleled increases in salinity in *Tetraselmis chui*, suggesting that it was involved in osmoregulation (*30*). Natural blooms of *Phaeocystis pouchetii* have endometabolomic profiles that include mannitol (*31*). The coccolithophore, *Chrysotila dentate*, has attached and free-living bacteria (mainly α-proteobacteria and γ-proteobacteria), which can use mannitol (*32*). *E. huxleyi* converts bicarbonate to carbon storage products such as mannitol (*33*). Different life stages of *E. huxleyi* show varying intracellular metabolites, and, for example, mannitol has been shown to have the highest intracellular concentration during exponential growth (*34*). The genes associated with metabolism of mannitol have been found in some haptophytes, but not all (*28*). Observations of the concentrations of mannitol in oceanic seawater have not been described to our knowledge.

### Glycerol osmotrophy

Glycerol plays a number of roles for marine microalgae. It can be effective for osmoregulation in the face of changing salinity due to its high water solubility and is also excreted from microalgae and microalgal symbionts (*35*). Glycerol can act as the sole carbon source for growth of coccolithophores in darkness for *E. huxleyi* and *Chrysotila carterae* (*36*). However, high concentrations were required for such growth (~0.5 M), orders of magnitude higher than would be expected in nature. Moreover, for those species, heterotrophic growth on high concentrations of glycerol has been shown to be sensitive to the total osmotic pressure of the medium and does not exceed an osmotic threshold (*36*). Blankley (*36*) showed that for two coccolithophore species (and 21 different strains tested) grown with glycerol, they could all grow indefinitely, through 5 to 20 transfers for 20 to 49 days in darkness with cell morphology identical for light- or dark-grown cultures [albeit a few of the clones produced fewer coccoliths in darkness and generation times were long (*E. huxleyi*, 6.4 to 8.6 days; *Pleurochrysis carterae*, 3.3 to 4 days)]. For medium without glycerol, cell growth, chlorophyll production, and calcite formation stopped after 2 days in darkness relative to light-grown cells. Production of chlorophyll a and particulate calcium (from PIC) continued for weeks when supplemented with glycerol, in both light- and dark-grown cells.

Heterotrophic growth of the haptophyte, *Prymnesium parvum*, in darkness on glycerol has also been documented (*37*). Other classes of microalgae (e.g. *Chlorella*, *Nannochloropsis*, *Rhodomonas*, *Porphyridium*, and *Cyclotella)* have been demonstrated to show increased yields when glycerol was presented as a carbon source and ammonia or nitrate was presented as a nitrogen source (*38*). Another study demonstrated enhanced growth on glycerol in an even wider range of microalgal genera: *Amphidinium*, *Karenia*, *Fugacium*, *Breviolum*, *Effrenium*, *Phaeodactylum*, *Emiliania*, *Phaeocystis*, *Dunaliella*, *Chlorella*, and *Rhodomonas* (*32*). Under nonaxenic conditions, 38% (11 of 29 species examined) were able to assimilate glycerol as a source of organic carbon. Fifty-two percent (15 of 29 species) showed no glycerol utilization. The remaining 10% of the species showed a negative or inhibitory effect of glycerol. Moreover, an examination of microalgal genomes and transcriptomes demonstrated that glycerol transport is widespread throughout the marine protists, present in 87% of the strains surveyed (*32*). As discussed above for mannitol, there are no systematic observations of glycerol concentrations from oceanic regions.

### Coccolithophores and the biological carbon pump and alkalinity pump paradigms

Carbon cycling by coccolithophores has been broadly related to the vertical carbon fluxes because this class of algae is fundamentally associated with two ocean “pump paradigms.” The first paradigm is the alkalinity pump (AP) (*39*) in which coccolithophore calcification lowers total alkalinity (TA) and dissolved inorganic carbon (DIC) of euphotic waters and produces CO_2_. When this calcium carbonate sinks into the deep sea with subsequent dissolution, surface alkalinity is effectively “pumped” to depth. The second pump paradigm is the biological carbon pump (BCP) in which any euphotic POC can be ballasted by aggregation with dense calcium carbonate coccoliths, effectively increasing the magnitude and/or transfer efficiency of the soft tissue (POC) flux to depth. The BCP also ultimately decreases surface CO_2_. The balance of the soft-tissue (POC) and CaCO_3_ (PIC) pumps influences whether the ocean is a net source or sink for CO_2_. The soft-tissue and APs reinforce each other in how they maintain a vertical gradient in DIC, but they oppose each other in terms of the air-sea exchange of CO_2_ (*40*). Thus, the net effect of coccolithophores on atmospheric CO_2_ depends on the balance of their CO_2_-raising effect associated with the AP and their CO_2_-lowering effect of promoting the soft-tissue BCP. It is virtually always assumed that the PIC found in coccoliths exclusively originates from DIC.

### Goals of this study

There were two goals of this study. The first goal was to measure the mixotrophic uptake and assimilation of ^14^C-acetate, ^14^C-mannitol, and ^14^C-glycerol as a carbon source by natural assemblages of coccolithophores and compare it to their autotrophic uptake and assimilation of DIC. These three organics were chosen because of their notable potential for osmotrophy by coccolithophores, as seen in previous culture studies. These compounds are also commercially available in radiolabeled form. The design of these experiments used radiochemical and single cell/flow cytometer (FCM) methods to distinguish osmotrophy of coccolithophores from that by other naturally occurring microalgae. The work was performed on a research cruise to the northwest (NW) Atlantic, a region known for abundant coccolithophore populations, either summer blooms near the Gulf of Maine (GOM) or less abundant but more diverse species assemblages observed offshore (*41*). The second goal of these studies was to test for the fixation of ^14^C-labeled organics into both POC and PIC fractions in natural populations of coccolithophores to examine the potential role of coccolithophore osmotrophy in the BCP and alkalinity carbon pump paradigms.

### Overview of experimental design

We achieved the above goals with the following experimental design. Briefly, we sampled nine stations in the NW Atlantic in July 2018, in search of locations and depths containing sufficiently large populations of coccolithophores to use in our experiments during four station occupations. The experiments involved incubating natural populations of phytoplankton with ^14^C-acetate, ^14^C-mannitol, ^14^C-glycerol, and ^14^C-bicarbonate (the latter of which was used to estimate photosynthetic carbon fixation), in separate vessels using simulated in situ conditions in a temperature-/light-controlled incubator over 24-hour incubations. Populations were harvested, either using bulk filtration of the entire phytoplankton community or by first concentrating the particulate matter using tangential-flow filtration, to sufficient densities that we could then sort only coccolithophores using an FCM. Next, we measured carbon fixation into POC and PIC fractions by measuring the ^14^C radioactivity using a high-sensitivity scintillation counter. To calculate uptake rates (in moles cell^−1^ day^−1^), we also needed to measure the concentrations of these three organic molecules in the original seawater samples (for which we devised the analytical methods to measure such low ambient concentrations found in seawater).

## RESULTS

The overall results of these experiments showed that, in eutrophic, mesotrophic, and oligotrophic waters of the NW Atlantic, in both coccolithophore bloom and nonbloom stations, we observed low ambient concentrations of the DOC compounds, acetate, mannitol, and glycerol. Moreover, we observed low rates of uptake of these compounds into both coccolithophore POC and PIC compared to photosynthetic fixation of bicarbonate. Fixation of DOC into PIC fractions was almost always higher when measured with bulk filtration than with FCM sorts, suggesting that the two methods were measuring different subsets of calcifiers.

### Hydrography and environmental conditions of the study area during EN616

**Fig. 1.**
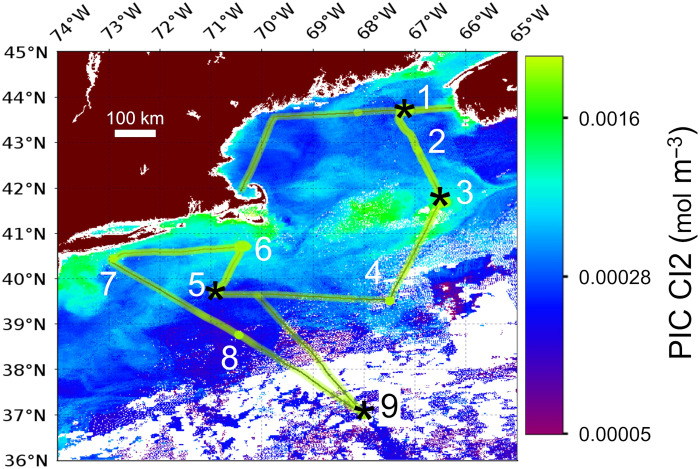
Locations of sampling stations in this study overlaid on an NPP/VIIRS satellite image of PIC concentration. Image from 8 July 2018 shows a coccolithophore bloom, which extended from the GOM to Mid-Atlantic Bight. PIC concentrations are keyed to the color scale at the right of the image (in units of mol PIC m^−3^). Organic uptake experiments using radiolabeled organic compounds and bicarbonate (experiments A, B, C, and D) were performed at stations 1, 3, 5, and 9, respectively (marked with asterisks). Stations 1, 2, 3, 6, and 7 were all located on the NE continental shelf. Stations 4, 5, and 8 were located on the continental slope. Station 9 was in the Sargasso Sea (NASA Ocean Color image file: SNPP_VIIRS-20180708.L3b.DAY.PIC). Scale bar, 100-km distance.

**Fig. 2.**
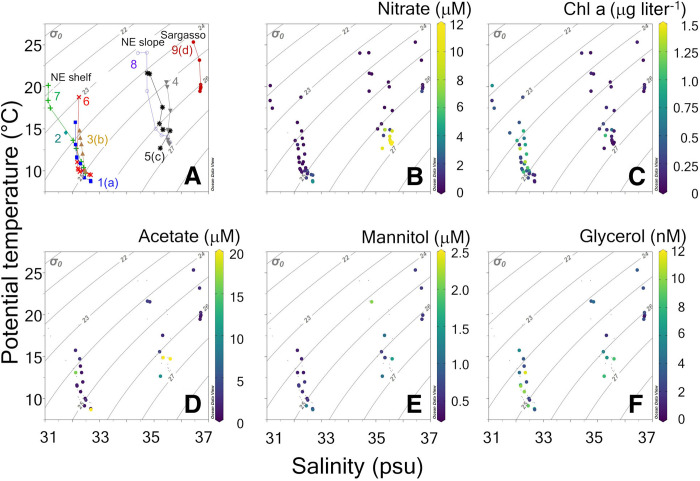
T/S diagrams for stations in study. (**A**) T/S diagram showing station numbers, designating general water masses for stations over the NE continental shelf, NE continental slope, and Sargasso Sea. Key to symbols designating each station profile: 1 (solid squares), 2 (solid diamonds), 3 (solid up-pointing triangles), 4 (solid down-pointing triangles), 5 (asterisks), 6 (X symbols), 7 (+ symbols), 8 (open circles), and 9 (solid circles). Also shown are concentrations of (**B**) nitrate (μM), (**C**) chlorophyll (Chl) a (μg liter^−1^), (**D**) acetate (μM), (**E**) mannitol (μM), and (**F**) glycerol (nM). Letters in brackets next to stations 1, 3, 5, and 9 designate experimental stations. (B) to (F) are keyed to color scale to right of each panel.

At the time of the cruise, there was a mesoscale coccolithophore bloom, predominantly consisting of the species *E. huxleyi* (but with at least 12 other coccolithophore species observed, based on scanning electron microscopy of the bloom assemblage), with the bloom centered over Georges Bank, at the southern flank of the GOM. The bloom was visible to polar-orbiting ocean color satellites (Fig. 1). At least 16 species of coccolithophores were observed at stations throughout the remainder of the study area. A more detailed description of the taxonomy of these waters will be published separately. A list of the stations visited in this study is given in Table 1. The general water mass description for the stations of the study are given as temperature/salinity (T/S) distributions, including data for concentrations of nitrate, chlorophyll a ambient acetate, mannitol, and glycerol (Fig. 2). A summary of the limits of detection, accuracy, precision, and workable range of the methods for measuring ambient acetate, mannitol, and glycerol (data presented in Fig. 2) can be found in table S1.

**Table 1.** Sample locations, dates, and times. Dates, positions, and time (GMT) for *Endeavor* cruise #616 are provided along with station and sample depth (in meters) from which water was collected for experiments A to D.

Station no.	Date (GMT)	Latitude (°N)	Longitude (°W)	Time (GMT)	Experiment no.	Sample depth for incubation experiment (m)
1	7/5/2018	43.717	67.209	1510	A	26
2	7/6/2018	42.477	66.948	1455		
3	7/7/2018	41.687	66.517	1458	B	12
4	7/8/2018	39.508	67.496	1447		
5	7/9/2018	39.696	70.901	1500	C	41
6	7/10/2018	40.709	70.292	1447		
7	7/11/2018	40.446	72.921	1439		
8	7/12/2018	38.759	70.455	1530		
9	7/13/2018	36.992	67.989	1457	D	131

The T/S measurements made at the nine stations of this study showed that three distinct water masses were sampled during the EN616 cruise: New England (NE) shelf waters, NE slope waters, and Sargasso Sea waters (Fig. 2). Shelf T/S plots resembled summertime (July to September) T/S distributions for the Great South Channel and Wilkinson basin, while slope water T/S distributions resembled summertime eastern slope water, previously described off the eastern flank of Georges Bank (*42*). The Sargasso Sea surface salinity of 36.3 practical salinity units (psu) resembled late summer surface values described previously in this same region (36.35 ± 0.05) (*43*).

On the basis of the chlorophyll concentrations (Fig. 2C), the shelf stations mostly classified as eutrophic (peak chlorophyll, >0.5 μg liter^−1^), slope waters as mostly mesotrophic (peak chlorophyll, 0.25 to 0.5 μg liter^−1^), and the one Sargasso Sea station as oligotrophic (chlorophyll, <0.25 μg liter^−1^) (*44*). The presence of measurable dissolved inorganic phosphorus (DIP) in the shelf and slope water column euphotic samples plus submicromolar nitrate in all but the deepest stations in these stations (fig. S1) suggest that overall productivity was nitrogen limited. Submicromolar ammonium concentrations also suggested that regenerated production was low (fig. S1) (*45*). Along with chlorophyll concentrations, POC, particulate organic nitrogen (PON), and PIC concentrations all showed most elevated concentrations in the shelf waters (fig. S2) with progressively lower concentrations in slope and Sargasso waters, respectively. Shelf stations demonstrated highest particle beam attenuation levels, lowest POC/PON ratios, and residual nitrate (nitrate-silicate) near zero (fig. S3). Highest concentrations of detached coccoliths and plated coccolithophores were found over Georges Bank in the coccolithophore bloom (station 3; fig. S4). When the vertical profiles were plotted as a function of the percent of surface light, there were subsurface chlorophyll maxima sitting over a deep nitracline, at approximately the 10% light level, for most stations (fig. S5). In short, as expected, the NE slope and Sargasso Sea stations were characterized by more nutrient stress than the NE shelf stations (with less phosphate and silicate in the surface layer, less ammonium, and lower chlorophyll biomass).

Ambient concentrations of acetate, mannitol, and glycerol showed highly variable profiles as a function of seawater density (Fig. 2). Acetate showed the highest ambient concentration of the three organic compounds, close to 20 μM. Profiles showed a subsurface maximum in NE slope waters, whereas in NE shelf waters, concentrations were maximal near the surface and near the bottom of the profiles, and in the Sargasso Sea, concentrations were uniformly undetectable, or a few micromolar, throughout the water column. Mannitol concentrations showed peak values just above 2 μM, and it was highly patchy as a function of seawater density, particularly at the NE slope stations. At the NE shelf and Sargasso Sea stations, low-density surface waters had mannitol concentrations below 1 μM, with a subsurface peak located near the sigma-theta 25 isopycnal. Mannitol concentrations in the Sargasso Sea were always less than 1 μM. Glycerol concentrations were the lowest of the three DOC compounds studied with peak concentrations of 11 nM. It also was highly patchy (Fig. 2).

Vertical distributions of acetate, mannitol, and glycerol showed no consistent pattern when plotted as a function of light (fig. S5). A more detailed description of the oceanographic setting is given in the Supplementary Materials (figs. S1 to S5).

### Initial conditions in incubation samples

The sampling conditions for each of the incubation experiments are given in Table 2 [temperature, salinity, light level (photosynthetic available radiation), percent surface light, concentrations of nutrients, POC, PON, PIC, chlorophyll, detached coccoliths, coccolithophores, and the specific DOC compounds targeted in these experiments]. Values of beam attenuation at 660 nm (in units of m^−1^) are also given. The conditions in our incubation experiments showed an overall temperature variability of ~9°C, salinity range of >4 psu, light intensities varying over two orders of magnitude, percent surface photosynthetically active radiation (PAR) from 0.1 to 8.5%, nitrate (0.12 to 1.83 μM), phosphate (0.05 to 0.29 μM), and silicate (0.1 to 1.60 μM) across all samples, plus POC:PON molar ratios of 7.75, 8.09, 5.38, and 14.05 for experiments A to D, respectively (Table 2). Ambient concentrations of acetate and mannitol were mostly submicromolar, and glycerol was at concentrations of 2 × 10^−9^ to 11 × 10^−9^ M (Table 2). For the incubations run at stations 1, 3, and 5 (experiments A to C, respectively), the samples came from above the nitracline (nitrate concentrations <0.3 μM), whereas for experiment D at station 9 in the Sargasso Sea, the nitrate concentration of 1.83 μM indicates that it came from below the nitracline. Of all the experiments, experiment A in the GOM showed the highest silicate levels (1.6 μM). The water sample for experiment B over Georges Bank (station 3; coccolithophore bloom) showed most-elevated concentrations of detached coccoliths and coccolithophore cells of all the experiments (~68,000 and ~400 particles ml^−1^, respectively). Of the three organics examined in this study, each showed peak concentrations in different experiments. For example, the highest acetate concentration of all the experiments was observed in experiment C (3.82 μM; mesotrophic station). The highest glycerol concentrations of all the experiments (11.5 nM) were found in experiment B from the coccolithophore bloom [and for this high concentration of glycerol, the sample had to be diluted for measurement (Table 2)]. The highest mannitol concentrations were observed in experiment D (2.25 μM) at the oligotrophic, Sargasso station 9 (Table 2).

**Table 2.** Starting environmental conditions for incubation samples. The beginning conditions of each incubation experiment are shown for temperature, light levels, concentrations of nutrients, POC, PON, PIC, chlorophyll a, detached coccoliths, plated coccolithophores, as well as average concentrations of acetate, glycerol, and mannitol (with SDs when available). Beam attenuation for the select depths is also provided.

Station no.	Experiment	Sample depth (m)	Temperature (°C)	Salinity	PAR at depth (μE m^−2^ s^−1^)	% Light	NO_3_ (μM)	PO_4_ (μM)	Si(OH)_4_ (μM)	NO_2_ (μM)	NH_3_ (μM)	POC (μmol liter^−1^)	PON (μmol liter^−1^)
1	A	26	10.84	32.30	55.12	2.36	0.12	0.29	1.60	0.02	0.21	34.12	4.40
3	B	12	13.11	32.33	205.29	8.54	0.25	0.15	0.40	0.02	0.89	30.41	3.76
5	C	41	15.61	35.20	62.59	2.68	0.16	0.05	0.50	0.00	0.00	14.27	2.65
9	D	131	19.50	36.68	2.51	0.11	1.83	0.05	0.10	0.01	0.11	2.81	0.20
**Station no.**		**Sample depth (m)**	**PIC (μmol liter** ^**−1**^ **)**	**Chlorophyll a (μg liter**^**−1**^)	**Beam attenuation (m**^**−1**^)	**Coccolithophore concentration (ml** ^**−1**^ **)**	**Coccolithophore concentration (ml** ^**−1**^ **)**	**Acetate average (μM)**	**Acetate SD (μM)**	**Glycerol average (nM)**	**Glycerol SD (nM)**	**Mannitol average (μM)**	**Mannitol SD (μM)**
1	A	26	0.3182	0.58	0.83	5,398	108	0.68	0.06	9.87	0.85	0.49	0.02
3	B	12	1.8812	0.87	1.07	68,083	424	0.81	0.33	11.50	2.10	0.37	0.03
5	C	41	0.4445	0.32	0.79	6,104	109	3.82		5.40		0.61	
9	D	131	0.4438	0.06	0.52	1,238	39	0.78	0.08	2.33	0.70	2.25	0.13

### Uptake of radiolabeled compounds

#### Raw counts

The first level of analysis of the data for uptake of the radiolabeled organic compounds was to examine the signal-to-noise ratio (SNR) for sample radioactivity [calculated as the mean and SD of 10 replicate counts in units of disintegrations per minute (DPMs) following the subtraction of killed blanks; table S2]. For each experiment and organic compound (plus ^14^C bicarbonate), mean radioactivity from the three incubations per compound was calculated after subtracting the respective formalin-killed blank for each radiolabeled compound and treatment. ^14^C-organic fixation by the bulk microbial population into the POC fraction (which includes all phytoplankton and bacterioplankton, not just coccolithophores) demonstrated that these organics were readily taken up by the microbial assemblages, with, in all cases, radioactivity counts >10,000 DPM, statistically significant above the killed control, based on Student’s *t* test (in all cases, degrees of freedom = 2; one tailed, *P* < 0.05; see table S2). There were a number of cases in experiments A, B, and C with the bulk POC radioactivity >100,000 DPM, significantly above the killed control for all the organics (table S2). The oligotrophic station 9 (experiment D) showed the lowest DPMs of the organics fixed into bulk POC but still statistically greater than the killed control, in the range of 8000 to 15,000 DPM (table S2). Bulk fixation of ^14^C-DOC into PIC represented fixation by coccolithophores but would have also included PIC in plated cells as well as detached coccoliths, empty coccospheres, or any other calcifying species that happened to be calcifying during the incubation (including a calcifying dinoflagellates, foraminifera, etc.). These bulk PIC counts were lower than the bulk POC counts, in all cases but one (experiment D, with ^14^C-bicarbonate in which there was more radioactivity found in the bulk PIC fraction than bulk POC fraction after the 24-hour incubation).

For the FCM sorts of coccolithophores, the signal of ^14^C-DOC fixed into POC and PIC fractions was lower than in the bulk fractions, but for POC fractions, the radioactivity signal was statistically greater than the formalin-killed blank about half the time (table S2). In this case, as opposed to the bulk filtrations, the FCM-sorted fractions contained POC DPMs associated only with coccolithophore POC, not other microbial populations. As with the bulk PIC results, the FCM sorts of coccolithophores showed statistically significant ^14^C-PIC signal originating from ^14^C-acetate (experiment C; table S2). Uptake of ^14^C-mannitol (experiment C) and ^14^C-glycerol (experiment A) was statistically significant, but only at a significance of *P* < 0.1, not *P* < 0.05. Among the ^14^C-DOC compounds, the radioactivity signal associated with the POC fraction in FCM sorts ranged from ~20 to >400 DPM higher than the killed control, in cases when it was statistically significant (table S2). For sorted coccolithophores, the signal of ^14^C-DOC fixed into PIC, when statistically greater than the killed control, fell in the range of 12 to 22 DPM (table S2). For ^14^C fixation of bicarbonate by coccolithophores, a statistically significant signal was observed in both POC and PIC in experiments A to C, with counts of 31 to 2700 DPM, statistically above the killed control (table S2; Student’s *t* test, *P* < 0.05).

### Cellular uptake rates

#### Concentrations of labeled compounds relative to total ambient concentrations

The fraction of radiolabeled DOC or DIC relative to the total DOC or DIC concentration (labeled and unlabeled) in each incubation experiment is shown in Table 3. Note that the ambient concentration of each organic was not known at the time of sampling since DOC concentrations were determined after cruise. For the DOC compounds, only two of the experiments had radiolabeled additions of DOC compounds less than 10% of the total concentration (experiment C for acetate and experiment D for mannitol), thus were considered true tracer experiments (*46*). Overall, for acetate, the fraction of the radiolabeled compound ranged from 5.7 to 25.5% of the total acetate. For mannitol, the radioisotopic form ranged from 8.4 to 35.9% of the total concentration, and for glycerol, the isotopic form ranged from 86.7 to 97.1% of the total (table S3). However, for bicarbonate, the fraction of isotope was always about three orders of magnitude below the total bicarbonate (table S3), thus were always true tracer experiments.

**Table 3.** Cellular assimilation rates of radiolabeled organic compounds. Assimilation rates of organic compounds and bicarbonate into coccolithophores were measured using either filtered, bulk seawater samples or FCM-sorted cells. Values represent the mean of three replicate incubation bags, each measured in triplicate. SDs of total samples represent not only the variability of the measurement technique (table S2) but also the variability between the three replicate incubations and their associated CV (SD divided by the mean). Assimilation of the radiolabeled compounds into PIC and POC fractions of FCM-sorted coccolithophores is shown. Only the cellular assimilation of radiolabeled compounds into bulk PIC fractions is shown. Bulk organic cellular assimilation rates are not presented since such rates are representative of all cells in the seawater sample, not just coccolithophores. The percentage of uptake of each organic compound relative to the total bicarbonate uptake is provided in four right-most columns.

		Bulk populations			FCM-sorted coccolithophores						Bulk		FCM		
Experiment	Radiolabeled	PIC	SD	CV (%)	POC	SD	CV (%)	PIC	SD	CV (%)	POC	PIC	POC	PIC	
	compound	[mol (coccolithophore cell)^−1^ day^−1^]			[mol (coccolithophore cell)^−1^ day^−1^]						% Activity incorporation of organic/bicarbonate				
A	^14^C-Acetate	1.77 × 10^−15^	9.40 × 10^−16^	53.2	–	–	–	1.97 × 10^−17^	2.16 × 10^−17^	109.6	0.064	0.030	–	0.001	
	^14^C Mannitol	1.05 × 10^−15^	2.01 × 10^−16^	19.2	2.80 × 10^−17^	6.27 × 10^−17^	224.2	3.71 × 10^−17^	2.71 × 10^−17^	72.9	0.054	0.018	0.001	0.003	
	^14^C-Glycerol	4.97 × 10^−17^	4.52 × 10^−17^	91.1	2.51 × 10^−17^	4.87 × 10^−17^	193.8	1.49 × 10^−17^	1.29 × 10^−17^	87.1	0.014	0.001	0.001	0.001	
	^14^C-HCO_3_^−^	5.87 × 10^−12^	1.31 × 10^−12^	22.3	2.52 × 10^−12^	1.65 × 10^−12^	65.6	1.36 × 10^−12^	1.13 × 10^−12^	83.0					
B	^14^C-Acetate	3.22 × 10^−16^	1.29 × 10^−16^	39.9	1.35 × 10^−16^	1.80 × 10^−17^	13.3	2.14 × 10^−18^	1.56 × 10^−16^	7287.0	0.158	0.005	0.035	0.001	
	^14^C Mannitol	3.14 × 10^−16^	1.18 × 10^−16^	37.6	3.44 × 10^−17^	3.64 × 10^−17^	105.8	–	–	–	0.241	0.004	0.009	–	
	^14^C-Glycerol	5.41 × 10^−20^	4.09 × 10^−20^	75.6	2.44 × 10^−18^	9.37 × 10^−19^	38.4	–	–	–	0.000	0.000	0.001	–	
	^14^C-HCO_3_^−^	7.08 × 10^−12^	4.70 × 10^−12^	66.4	3.92 × 10^−13^	7.31 × 10^−14^	18.7	3.51 × 10^−13^	5.66 × 10^−14^	16.1					
C	^14^C-Acetate	1.58 × 10^−14^	2.19 × 10^−15^	13.9	7.25 × 10^−16^	1.79 × 10^−16^	24.7	4.98 × 10^−16^	4.63 × 10^−16^	93.0	0.955	0.911	0.260	4.154	
	^14^C Mannitol	1.78 × 10^−15^	3.39 × 10^−16^	19.0	1.29 × 10^−15^	1.68 × 10^−15^	129.7	3.95 × 10^−17^	3.72 × 10^−17^	94.1	0.851	0.103	0.463	0.330	
	^14^C-Glycerol	6.92 × 10^−17^	2.96 × 10^−17^	42.8	4.04 × 10^−17^	2.72 × 10^−17^	67.3	1.90 × 10^−19^	2.53 × 10^−18^	1332.7	0.210	0.004	0.014	0.002	
	^14^C-HCO_3_^−^	1.74 × 10^−12^	2.18 × 10^−13^	12.6	2.79 × 10^−13^	1.36 × 10^−13^	48.6	1.20 × 10^−14^	2.06 × 10^−14^	171.6					
D	^14^C-Acetate	1.52 × 10^−14^	9.89 × 10^−15^	65.1	6.97 × 10^−17^	3.88 × 10^−17^	55.7	5.86 × 10^−18^	3.60 × 10^−18^	61.4	–	0.042	2.545	–	
	^14^C Mannitol	4.33 × 10^−15^	4.66 × 10^−15^	107.5	6.49 × 10^−17^	1.75 × 10^−17^	26.9	–	–	–	–	0.012	2.369	–	
	^14^C-Glycerol	1.84 × 10^−16^	2.61 × 10^−17^	14.2	5.15 × 10^−18^	1.74 × 10^−18^	33.8	–	–	–	–	0.001	0.188	–	
	^14^C-HCO_3_^−^	3.62 × 10^−11^	2.81 × 10^−12^	7.8	2.74 × 10^−15^	1.85 × 10^−15^	67.5	–	–	–					

### Bulk measurements

**Fig. 3.**
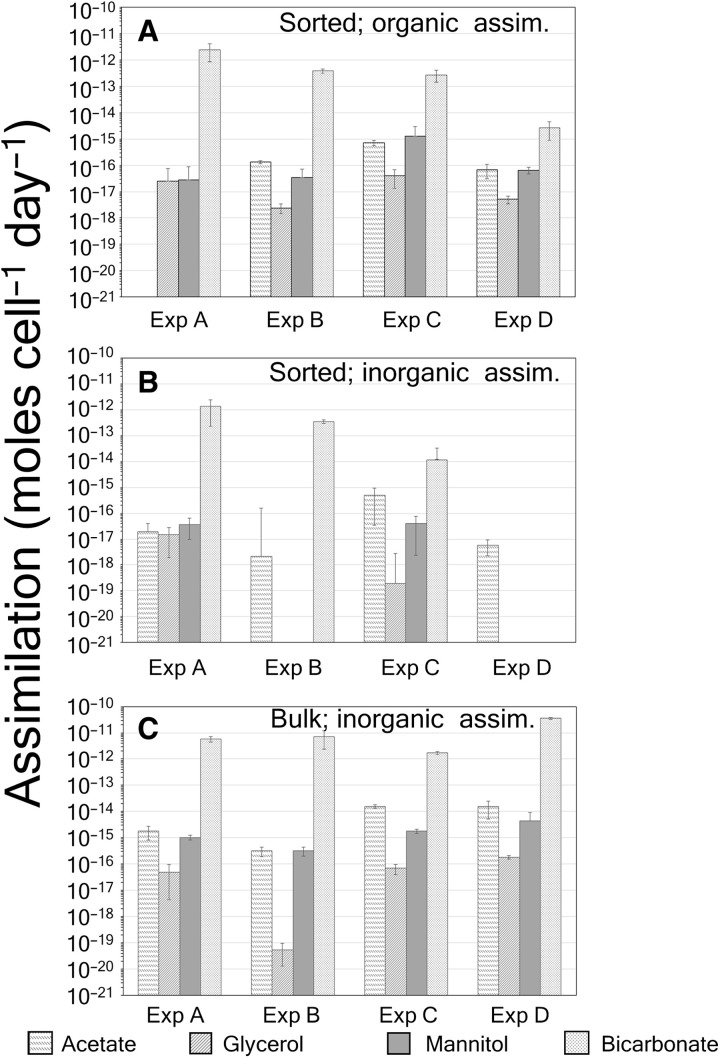
Cellular assimilation rates of three ^14^C-labeled organic compounds as well as ^14^C-bicarbonate. (**A**) Into POC of FCM-sorted coccolithophores, (**B**) into PIC of sorted coccolithophores, and (**C**) bulk PIC of all calcifying organisms (not necessarily just coccolithophores) normalized to coccolithophore concentration. Histograms of cellular uptake rates with SDs of triplicate measurements are shown for ^14^C-acetate, ^14^C-glycerol, ^14^C-mannitol, and ^14^C-bicarbonate. Cell concentrations of coccolithophores used to calculate cellular rates were based on birefringence microscopy for bulk samples and FCM cell counts for the FCM-sorted samples.

The results of the measurements of incorporation of ^14^C-DOC into bulk PIC fractions are given in Table 3 and Fig. 3C along with SDs for triplicate measurements from the same water sample. These samples provided a cross-check on the rates estimated with FCM-sorted cells. Note that no estimate of cellular incorporation of ^14^C-DOC into coccolithophore POC was possible with bulk filtrations since the bulk filtrations sampled more DOC-fixing microbes than just coccolithophores. However, the ^14^C-PIC caught on the bulk filters most likely originated from coccolithophore cells (and possibly other calcifiers like foraminifera, except that they are orders of magnitude less abundant in seawater and less likely to be caught in our <1-liter sample volumes). The bulk cellular rates of uptake were calculated by normalizing the uptake rates by the coccolithophore concentration (derived from quantitative birefringence counts performed after cruise), whereas the calculated rates from the FCM sorts were derived directly from the sorts.

**Fig. 4.**
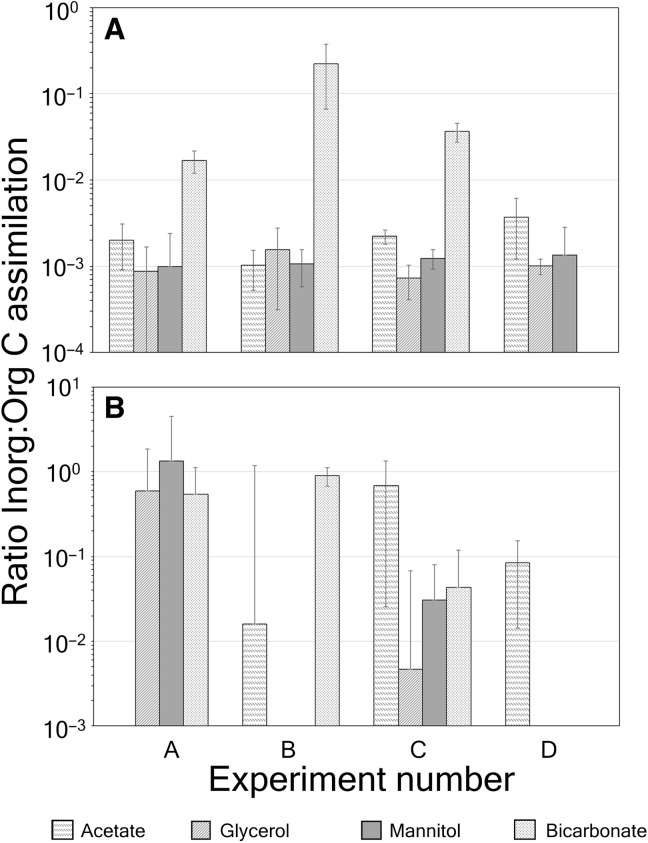
Ratio of fixation of radiolabeled compounds into inorganic and organic fractions for experiments A to D. (**A**) Bulk samples and (**B**) for FCM-sorted samples. Error bars are SDs for triplicate incubations (including SE propagation for ratios).

The highest coccolithophore-specific uptake rates of organics into bulk PIC were for acetate [15.8 × 10^−15^ mol acetate (coccolithophore cell)^−1^ day^−1^] in experiment C (slope station) (Fig. 3C). More typical fixation rates of ^14^C-DOC into bulk PIC were ~0.1 × 10^−15^ to 10 × 10^−15^ mol cell^−1^ day^−1^. A comparison of these rates to the fixation of bicarbonate into PIC shows that the rate of incorporation of ^14^C-DOC into coccolithophore PIC was low, typically 0.001 to 0.03% of the rate of bicarbonate fixation into PIC (Table 3). Note that in experiment C, acetate and mannitol incorporation into PIC was 0.91 and 0.10% of the rate of bicarbonate incorporation, respectively (Table 3). As noted above, cell-specific rates of incorporation of ^14^C-DOC into POC could not be calculated with the bulk samples due to lack of information on the total number of cells of all microalgae responsible for the uptake, not just coccolithophores. However, the fraction of the ^14^C-DOC uptake into total POC could still be compared with the total ^14^C-bicarbonate uptake into POC. No uptake of ^14^C-DOC into POC was detected in the bulk samples from the Sargasso Sea experiment D (table S3). For the mesotrophic and eutrophic stations (experiments A to C), the ^14^C-DOC uptake rates into bulk POC represented 0.01 to 0.95% of the bicarbonate uptake into POC (Table 3). Overall, for the fixation of ^14^C-DOC into PIC or POC, there was high variability between the replicate bulk measurements, with coefficients of variation (CVs) (SD/mean) of ~10 to 100% (Table 3). For all the bulk filtration samples, the ratio of fixation of ^14^C-DOC into PIC versus POC was roughly 0.1 to 0.3% (Fig. 4A), whereas for bicarbonate fixation, the inorganic:organic ratio was ~1.7% (experiment A), 22% inside the Georges Bank coccolithophore bloom (experiment B), 3.7% for the NE slope waters (experiment C), and 0% in the Sargasso Sea (experiment D) (Fig. 4A).

### Cellular rates of incorporation based on FCM-sorted coccolithophores

Incorporation of ^14^C-organics into PIC of the FCM-sorted cells varied from 10^−19^ to 10^−16^ mol cell^−1^ day^−1^, while fixation of bicarbonate into coccolithophore PIC was 10^−14^ to 10^−12^ mol cell^−1^ day^−1^ (Fig. 3B). The fixation of ^14^C-DOC into PIC was <0.003% of the total bicarbonate uptake for experiments A and B (Table 3). For experiment C, acetate, mannitol, and glycerol uptake showed elevated incorporation rates into PIC, representing 4.15, 0.33, and 0.002% of the ^14^C-bicarbonate taken up into PIC, respectively (Table 3). For experiment D, uptake of acetate into PIC was exceedingly low and barely detectable based on replicate measurements (Table 3). Uptake of mannitol and glycerol by coccolithophores into their calcite coccoliths was not statistically significant nor was there any measurable bicarbonate uptake into PIC either (Table 3).

FCM-sorted cells allowed the same calculations to be made for the incorporation of ^14^C-DOC into POC. Across the three DOC compounds, cell-specific rates of incorporation into POC were between 10^−18^ and 10^−15^ mol cell^−1^ day^−1^ (Fig. 3A), which were 0.001 to 2.5% of the total ^14^C-bicarbonate activity fixed into POC (Table 3). The CVs of the rates of ^14^C-DOC incorporated, as determined with FCM-sorted cells, were higher than for the bulk filtrations, presumably due to lower sample size and the lower signal to noise (Table 3 and fig. S2).

### PIC:POC ratios of assimilated carbon

For the bulk filtrations, the ratio of ^14^C-DOC fixed into PIC versus POC varied from 0.07 to 0.37% (Fig. 4A). For bulk samples, the ratio of ^14^C-bicarbonate fixed into PIC versus POC was ~1.7, 22.1, and 3.7% for experiments A to C, respectively, a larger fraction than for the assimilation of the DOC compounds. For experiment D (Sargasso Sea), there was no measurable bicarbonate uptake into PIC (Fig. 4). For the FCM-sorted cells, in the 10 cases when there was measurable uptake of ^14^C-bicarbonate or ^14^C-DOC, for six of those, the PIC:POC ratios of the assimilated ^14^C were between 0.5 and 1, and all but one exceeded a ratio of 0.01 (Fig. 4B). These ratios were far higher than the ratios for the bulk filtrations above (Fig. 4A).

**Fig. 5.**
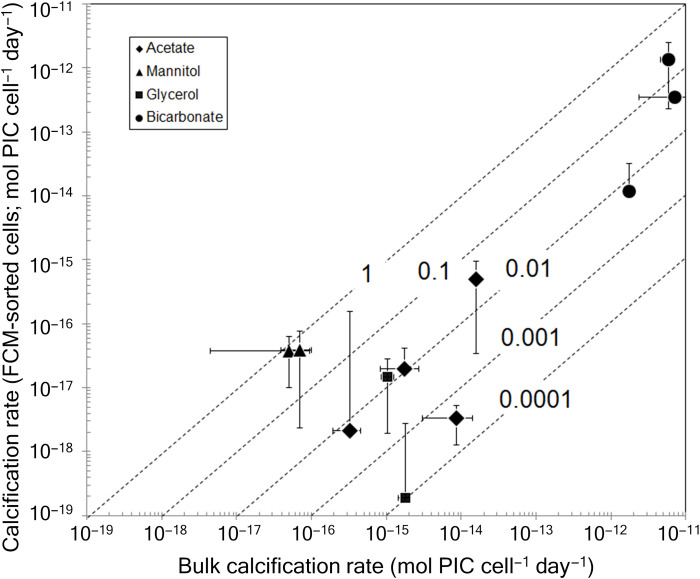
Cellular calcification rates estimated from FCM-sorted and bulk-filtered samples. Mean bulk calcification rates calculated by dividing the bulk-derived calcification by the concentration of coccolithophores (estimated using bulk counts of birefringent coccolithophores). Error bars represent one SD about the means. Diagonal lines show different order-of-magnitude ratios of FCM-derived cellular calcification rates to bulk-derived cellular calcification rates.

A comparison of cellular assimilation rates of the four radiolabeled compounds into PIC is shown for FCM-sorted cells versus bulk-filtered cells. The results reveal that for most of the ^14^C-DOC compounds, the FCM-derived fixation rates into PIC were equal to or less than the bulk-derived rates, sometimes by about four orders of magnitude. For bicarbonate fixation, the FCM-derived cellular fixation rates were ~1 to about 30% of the bulk-derived rates (Fig. 5).

## DISCUSSION

In this study, we have demonstrated osmotrophy in natural assemblages of coccolithophores. Moreover, we have also shown that rapid uptake of DOC (three specific compounds: acetate, mannitol, and glycerol) can be traced not only into the soft tissues of coccolithophores but also into calcium carbonate coccoliths. Below, we elaborate on these results and compare the observations across the different ocean regions and to results from other published studies. Last, we address the implications of this work to overall coccolithophore nutrition as well as the ocean biological and AP paradigms.

### Comparison of cellular photosynthesis and calcification rates by coccolithophores using ^14^C-bicarbonate

We compared our photosynthesis and calcification rate results (Table 3) with other published studies for the estimates using ^14^C bicarbonate as a substrate (Table 4). Bulk measurements of cellular photosynthesis were not possible with our natural assemblages because we did not enumerate cell abundance of all classes of algae but instead enumerated only the coccolithophores. Nonetheless, for previous published coccolithophore culture studies, bulk measurements of productivity could be predicted using the product of POC cell quota (POC cell^−1^) and cellular growth rate (day^−1^) (*47*, *48*). Such estimates of coccolithophore photosynthesis fell within the range of 10^−15^ to 10^−12^ moles cell^−1^ day^−1^ (Table 4). The estimates of photosynthesis made using the FCM-sorted coccolithophores from field assemblages reported here fell in a similar range (Table 4).

**Table 4.** Cellular uptake rate comparison. Comparison of coccolithophore bicarbonate and organic uptake rates from this study with estimates from the literature, shown for fixation into POC and PIC fractions, made using either sampling techniques of bulk filtration or FCM sorting.

Category/sampling	Reference no.	Substrate	Coccolithophore species	mol cell^−1^ day^−1^
Particulate organic C				
Bulk filtration	(*47*)*	HCO_3_^−^	*Coccolithus pelagicus*	4.42 × 10^−12^ to 8.0 × 10^−12^
	(*47*)*	HCO_3_^−^	*E. huxleyi* (RCC1228)	0.34 × 10^−12^ to 0.51 × 10^−12^
	(*48*)*^,†^	HCO_3_^−^	9 different coccolithophore strains	0.059 × 10^−12^ to 1.91 × 10^−12^
	(*17*)^‡^	Acetate	*Cruciplacolithus neohelis* (CCMP298)	0.2 × 10^−12^
	(*17*)^‡^	Acetate	*C. carterae* (CCMP3337)	1.2 × 10^−12^
	(*17*)^‡^	Mannitol	*C. neohelis* (CCMP298)	0.1 × 10^−12^
	(*17*)^‡^	Mannitol	*C. carterae* (CCMP3337)	0.1 × 10^−12^
	(*17*)^‡^	Glycerol	*C. neohelis* (CCMP298)	0.25 × 10^−12^
	(*17*)^‡^	Glycerol	*C. carterae* (CCMP3337)	0.15 × 10^−12^
	(*36*)*^§^*	Glycerol	*C. carterae* (P-156)	0.21 × 10^−12^
FCM-sorted	This study	HCO_3_^−^	Natural assemblage	2.74 × 10^−15^ to 2.52 × 10^−12^
	This study	Acetate	Natural assemblage	0 to 0.725 × 10^−15^
	This study	Mannitol	Natural assemblage	28.0 × 10^−18^ to 1.29 × 10^−15^
	This study	Glycerol	Natural assemblage	2.44 × 10^−18^ to 40.4 × 10^−18^
Particulate inorganic C				
Bulk filtration	This study	HCO_3_^−^	Natural assemblage	1.7 × 10^−12^ to 36.2 × 10^−12^
	(*47*)*	HCO_3_^−^	*C. pelagicus*	5.31 × 10^−12^ to 9.63 × 10^−12^
	(*47*)*	HCO_3_^−^	*E. huxleyi* (RCC1228)	0.26 × 10^−12^ to 0.38 × 10^−12^
	(*48*)*^,†^	HCO_3_^−^	9 different coccolithophore strains	0.06 × 10^−12^ to 2.68 × 10^−12^
	(*17*)*^ǁ^*	Acetate	*C. neohelis* (CCMP298)	2 × 10^−15^
	(*17*)*^ǁ^*	Acetate	*C. carterae* (CCMP3337)	5 × 10^−15^
	(*17*)*^ǁ^*	Mannitol	*C. neohelis* (CCMP298)	7 × 10^−16^
	(*17*)*^ǁ^*	Mannitol	*C. carterae* (CCMP3337)	3 × 10^−15^
	(*17*)*^ǁ^*	Glycerol	*C. neohelis* (CCMP298)	6 × 10^−17^
	(*17**)^ǁ^*	Glycerol	*C. carterae* (CCMP3337)	7 × 10^−18^
	(*36*)^§^	Glycerol	*C. carterae* (P-156)	0.595 × 10^−12^
	This study	Acetate	Natural assemblage	0.322 × 10^−15^ to 15.8 × 10^−15^
	This study	Mannitol	Natural assemblage	0.314 × 10^−18^ to 4.33 × 10^−15^
	This study	Glycerol	Natural assemblage	5.41 × 10^−20^ to 0.184 × 10^−15^
FCM-sorted	This study	HCO_3_^−^	Natural assemblage	0 to 1.36 × 10^−12^
	(*49*)	HCO_3_^−^	*E. huxleyi*	0.6 × 10^−12^
	This study	Acetate	Natural assemblage	2.14 × 10^−18^ to 0.498 × 10^−15^
	This study	Mannitol	Natural assemblage	0 to 39.5 × 10^−18^
	This study	Glycerol	Natural assemblage	0 to 14.9 × 10^−18^

*Calculated from the average measured growth rate (0.17 day^−1^) multiplied by POC cell quota for dark-grown cells *(**17**)* [as based on cell size and the equations of Villiot *et al.* (*48*)]. Calcification estimates based on figure IV.36 of Blankley (*36*) and multiplying the above growth rate times the Ca cell quota at 650 hours (140 pg Ca cell^−1^ = 3.5 pmol cell^−1^).

†Calculated from the POC or PIC cell quota of nine species of coccolithophores multiplied by an assumed growth rate of 0.5 day^−1^ (*48*). These species included *Reticulofenestra parvula* (RCC 4036), *E. huxleyi* (RCC 1731), *E. huxleyi* (RCC 1228), *Gephyrocapsa muellerae* (RCC 3370), *Gephyrocapsa oceanica* (RCC1314), *C. leptoporus* (RCC 1130), *Syracosphaera pulchra* (RCC 1461), *C. leptoporus* (RCC 1135), and *Coccolithus braarudii* (RCC 1198).

‡Calculated from bulk measurements of cultures multiplied by cellular growth rate.

§Taken from *V*_max_ values for incorporation of organic molecules into PIC from figure 5 of Godrijan *et al.* (*17*).

ǁTaken from *V*_max_ values for incorporation of organic molecules into PIC, from figure 6 of Godrijan *et al.* (*17*).

Cellular calcification rates measured in bulk in this study (with normalization to bulk counts of birefringent coccolithophores) generally fell into the range of 2 × 10^−12^ to 36 × 10^−12^ moles cell^−1^ day^−1^. Rates of bicarbonate uptake into PIC were similar to estimates based on the product of PIC cell quota and growth rate (*47*, *48*) with maximum values of 2.7 × 10^−12^ to 9.6 × 10^−12^ moles cell^−1^ day^−1^ (Table 4). However, the calcification rates based on FCM-sorted coccolithophores from natural assemblages were lower than the bulk rates, showing maximal values of only 1.4 × 10^−12^ moles cell^−1^ day^−1^ (Fig. 5). Moreover, these were slightly higher than one previously published, FCM-sorted, calcification rate for *E. huxleyi* [0.6 × 10^−12^ moles cell^−1^ day^−1^ (*49*), which, itself, was well within the range of other bulk estimates of bicarbonate assimilation into PIC; Table 4]. One possible reason for the low calcification rate for our natural, FCM-sorted coccolithophore assemblages is the narrow range of side scatter, fluorescence, and birefringence gating used to key in on natural coccolithophores. The FCM gates were set using a culture of *Crucioplacolithus neohelis* (CCMP298) that was brought to sea (with which we had considerable previous experience in the laboratory). It is possible that the gating used to optimize the sorting of the natural populations simply keyed the cell sorts to similar small coccolithophore species, which may have predisposed the field sorts to small cells with low cellular calcification rates. Moreover, there were many other birefringent particles (suggestive of CaCO_3_ composition) that contained no red chlorophyll fluorescence (fig. S6). These were probably detached coccoliths or empty coccospheres. Since they were outside the FCM gates, they would not have been sorted and, hence, could also have caused underestimates in the FCM-derived calcification rates when compared to the bulk measurements.

### Assimilation rates of acetate, mannitol, and glycerol compared to other published rates

As with bicarbonate uptake above, we compared our measured assimilation rates of organic molecules into POC and PIC fractions to uptake rates published in two previous studies (Table 4) (*17*, *36*). For example, incorporation of acetate, mannitol, and glycerol into coccolithophore POC, as measured in other reports, mostly fell in the range of hundreds of femtomoles cell^−1^ day^−1^ to picomoles cell^−1^ day^−1^ (Table 4) (*17*). On the other hand, FCM-sorted, natural assemblages of coccolithophores measured in our ship experiments showed maximum rates of incorporation of acetate and mannitol into POC of 10^−15^ moles cell^−1^ day^−1^ (Table 4). Maximum incorporation of glycerol into the POC of natural coccolithophore assemblages was far lower at 40 × 10^−18^ moles cell^−1^ day^−1^ (for FCM-sorted cells).

Maximal rates of incorporation of these organic compounds into cultured coccolithophore PIC were previously shown by Godrijan *et al.* (*17*) to be several femtomoles cell^−1^ day^−1^ when measured in bulk. Cellular rates of incorporation of the same organic compounds by natural coccolithophore assemblages into PIC were in a similar range for the bulk measurements and less for the sorted samples (Table 4). Blankley (*36*) measured calcium incorporation (i.e., calcification; see his figure IV.36). Multiplying his *C. carterae* growth rate (0.17 day^−1^) by the measured Ca cell quota (3.5 × 10^−12^ moles/cell) gave a calcification rate of 0.595 × 10^−12^ mol cell^−1^ day^−1^ after 650 hours in darkness, well above the rates cited here. However, these rates were measured following exposure to far higher ambient concentrations of glycerol (0.2 to 0.5 M, as compared to our experiments in which the glycerol concentration was orders of magnitude lower, 2.33 to 11.5 μM; Table 2), which likely would have increased the cellular rates of diffusion/uptake and, ultimately, assimilation in Blankely’s experiments (*36*). The high observed variability in organic uptake observed in the natural assemblages of coccolithophores shown here likely also resulted from a wide variety of species in those natural populations combined with growth physiology limited by various other rate-limiting constituents and ambient concentrations of the substrates that were orders of magnitude lower in these field experiments than in previous laboratory experiments.

### Experimental errors and limitations

For all but one of the experiments (experiment D for mannitol uptake), the addition of the radiolabeled DOC tracers did not qualify as true trace additions relative to the ambient concentrations [i.e., where the ^14^C-labeled form was <10% of the total concentration of labeled and ambient compound (*46*)]. The potential ramification of this is that if the transport of these DOC compounds into microalgal cells was facultative (induced by the increased concentration of the compound), then the introduction of the isotope may have stimulated the uptake and assimilation pathways of the compounds. This issue would have been greatest for the glycerol uptake experiments where the isotope contributed around 90% of the total concentration (table S3). On the other hand, radiolabeled bicarbonate additions were orders of magnitude below 1% of ambient bicarbonate concentrations and can be considered true tracers in these experiments (table S3).

For the bulk filtrations, the levels of radioactivity in the PIC fractions were high enough that errors in scintillation counting were far below the interreplicate errors. For example, the CV for all scintillation counts averaged ±3.7% for 10 replicate counts of each vial for all treatments, bulk or sorted (table S2). However, the average CV for rates derived from bulk filtrations (triplicate measurements from each bag) was ±32%, and for FCM sorts, the average CV was ±53%. The higher CV for the triplicate bags was probably due to the overall lower signal to noise of the FCM sorts, combined with the low cellular biomass (Table 3).

There was a notable disparity in cellular calcification rates between bulk-derived versus FCM-derived rates; the bulk rates showed orders of magnitude higher calcification rates than the FCM-derived rates (compare Fig. 3, B and C). This might have resulted because of two reasons. First, the coccolithophores targeted by the FCM were likely not the only calcifiers in the natural assemblage, and there were numbers of rare, large calcifiers captured in the bulk samples (not necessarily just coccolithophores but also calcifying dinoflagellates, foraminifera, etc.) that were not included in the FCM-sorted samples. That is, large, rare calcifiers might not have appeared in the FCM sorts due to their low overall concentration but would have been more likely to be present in the bulk samples. Such large, rare calcifiers would have had higher cell calcite quotas and higher cell-specific calcification rates. However, the abundances of foraminifera are so low compared to coccolithophores (there might be a few foraminifera per m^3^ compared to tens to hundreds of coccolithophores mL^−1^) that catching a foram on a bulk filter would have been possible, but unlikely. It would have been more likely that a few, rare, large coccolithophores were caught on the bulk filter but not captured by the FCM (*47*). Second, detached coccoliths and empty coccospheres would not have been sorted by the FCM since they were not fluorescent, but these would have been present in the bulk samples, leading to higher derived calcification rates.

To our knowledge, the vertical profiles of ambient acetate, mannitol, and glycerol concentration reported here represent some of the first such measurements of these organics in surface oceanic waters [as opposed to sedimentary pore waters (*27*)]. Nonetheless, analytical errors in ambient acetate, mannitol, and glycerol were another potential source of error between different incubation experiments; the measurements showed average CVs of 20, 19, and 5%, respectively (Table 2). Thus, errors in uptake rates associated with the DOC analyses were quantifiable but still less than the within-experiment replication error in ^14^C uptake measurements cited above (Table 3). Moreover, there would have been no difference in the DOC concentration in water used within a given incubation experiment, across all treatments, since they were all started from the same water sample.

### Potential environmental factors affecting osmotrophic uptake by coccolithophores

For the four experiments, bulk DOC uptake rates (which had greater signal to noise than the FCM uptake rates) of mannitol and glycerol into PIC were inversely correlated with optical beam attenuation of the original sample and positively correlated with sample depth. Both beam attenuation and sample depth varied along the continuum of eutrophy from the shallow, turbid station over Georges Bank to the deep, clearest station in the Sargasso Sea (see linear bivariate least squares regression statistics in table S4). The exception to this was acetate uptake rates, which were not significantly correlated with either beam attenuation or water depth for the bulk filtrations. In short, highest cellular rates of uptake of DOC (>1 × 10^−15^ mol cell^−1^ day^−1^) were not observed in the Georges Bank coccolithophore bloom (station 3), the region with the highest coccolithophore concentration. Instead, the highest bulk DOC fixation rates into coccolithophore PIC were observed for stations with lower overall turbidity and from greater depths [station 1 (experiment A; NE shelf-GOM; peak cellular uptake rates of mannitol and glycerol), station 5 (experiment C; NE slope water; peak cellular uptake rates of mannitol), and station 9 (experiment D; oligotrophic Sargasso Sea; peak cellular uptake rates of acetate and mannitol); Table 3]. In addition, qualitatively speaking, elevated DOC uptake rates into coccolithophore POC or PIC typically were seen for only one or two of the DOC compounds, not all three, and at rates that were about 10× to 100× faster than those observed in the high-density coccolithophore bloom over Georges Bank. This suggests that the transporters for these DOC compounds are selective for specific compounds and not generic for all DOC compounds. In contrast, the Georges Bank coccolithophore bloom was characterized by the highest cellular bicarbonate uptake rates via photosynthesis and calcification, whether for bulk or FCM-sorted coccolithophores (Table 3). Thus, these results would suggest that in the more meso- and oligotrophic environments, the osmotrophic strategy could play a more important role in overall coccolithophore nutrition than in a shallow coccolithophore bloom on Georges Bank. We caution, however, that DOC uptake rates in these four experiments showed no statistical correlation to initial concentrations of any of the nutrients (nitrate, nitrite, ammonium, phosphate, or silicate) or initial concentrations of chlorophyll, POC, and C/chlorophyll ratio, and thus, conclusions should be tempered accordingly.

### Implications of osmotrophy for coccolithophore growth and calcification

With the results shown here for just three types of DOC, it is curious whether, with the wide variety of DOC compounds in seawater (*50*), more DOC could be available to coccolithophores for osmotrophic growth. In the GOM, the concentration of total DOC is typically 0.8 to 1.3 mg C liter^−1^ (66.6 to 110 μmol C liter^−1^) (*51*); thus, the concentrations of organic molecules evaluated here (table S3) represent only a few percent of the total DOC compounds present in these waters. Twenty-five varieties of cultured coccolithophores (including 12 species) were able to take up as many as 19 of 32 (59%) of the DOC compounds presented to them at low irradiance levels (*16*). This would suggest that osmotrophy has the potential to be a mode of coccolithophore nutrition, especially in low-light conditions found in sub-euphotic populations, or even historically, following the Cretaceous-Tertiary boundary asteroid impact when sunlight was blocked for long periods of time (*17*). A similar argument has been advanced for survival and recovery of phagotrophic coccolithophores following the end-Cretaceous asteroid impact (*52*). However, it is unlikely that coccolithophores could sustain elevated growth rates based solely on an osmotrophic strategy but, more likely, slow growth rates that allow survival, consistent with previous laboratory experiments (*17*). Given our field results, there is also the potential that a larger fraction of PIC could be contributed by DOC assimilation, as well. However, evidence for this using field populations has yet to be demonstrated. More field research is needed using sensitive isotope tracer experiments to examine the uptake/assimilation of other labeled DOC compounds, or classes of compounds, to understand the broader contribution of DOC to PIC production by coccolithophores.

### Implications to the biological pump and AP paradigms

**Fig. 6.**
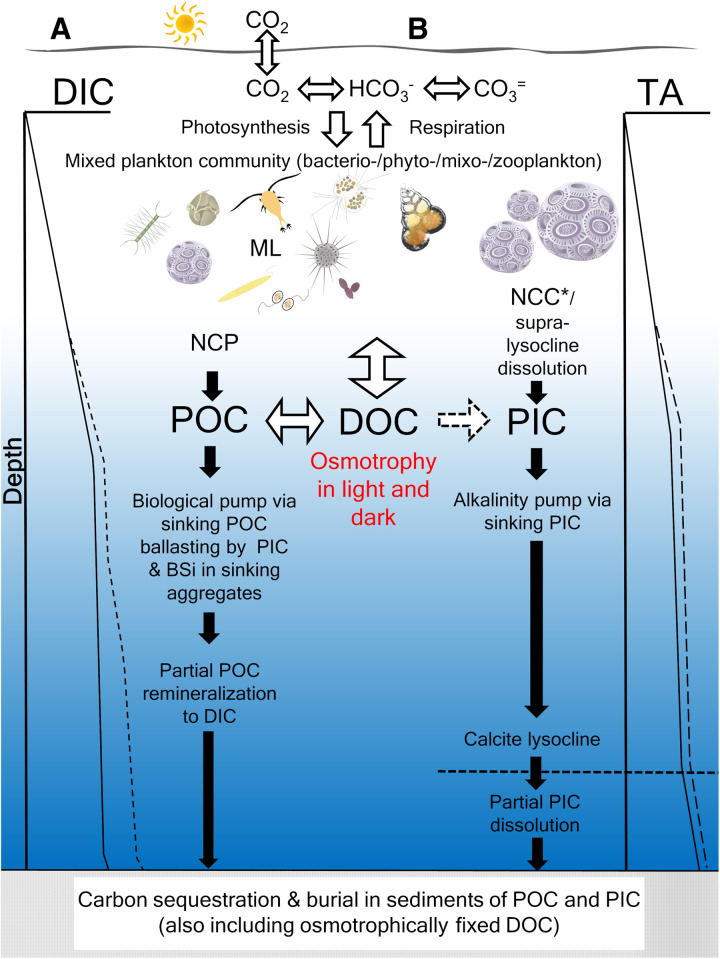
Conceptual model on connections between coccolithophore osmotrophy and BCP/AP paradigms. (**A**) BCP (left)—The BCP integrates net community production and sinking POC flux from the mixed planktonic community. Photosynthesis consumes CO_2_ and HCO_3_^−^ with the CO_2_ in equilibrium with atmospheric CO_2_. Coccolithophore PIC and diatom BSi provide ballast for sinking POC, accelerating sinking rates. PIC buffers sinking aggregates, protecting POC from remineralization, enhancing downward transport. Many algae are osmotrophic (including coccolithophores), thus fixing DOC into sinking POC. Left DIC profile shows net DIC drawdown by algae. Dashed line illustrates additional DIC resulting from remineralized POC, originally associated with DOC. Bidirectional arrow between DOC and POC illustrates the community assemblage releasing and consuming DOC. “ML” represents the microbial loop of DOC and POC between marine bacterio-/phyto-/mixo-/zoo-plankton. The BCP sequesters POC, PIC, and DOC into sinking aggregates. Partial or complete remineralization of POC to DIC occurs during aggregate descent, increasing DIC concentration. (**B**) AP (right)—Coccolithophore calcification reduces bicarbonate/carbonate alkalinity in surface waters, fixing carbon into sinking CaCO_3_, driving the AP. NCC represents all calcifiers (e.g., coccolithophores, foraminifera, and pteropods); we refer to coccolithophore calcification/dissolution only as NCC*. A one-directional arrow connects DOC and PIC pools, since, when that PIC dissolves, it releases DIC as bicarbonate, not DOC. DOC fixation into PIC is likely mediated by marine bacteria and/or the algae that consume DOC [which, depending on the molecule, may or may not contribute organic alkalinity (*59*), and metabolize it to DIC that coccolithophores take up]. The calcite lysocline marks an increase in dissolution during sinking, thus releasing bicarbonate (originally from surface waters) at depth. Right-hand vertical profile illustrates TA with surface drawdown from calcification and deeper increases from PIC dissolution. Dashed line represents additional TA resulting from DOC fixed into PIC and subsequently dissolved at depth to bicarbonate.

These results have implications to the traditional BCP and AP paradigms (see conceptual model in Fig. 6). The BCP paradigm assumes that sinking particulate organic matter to deep sediments is, in part, ballasted by associated biogenic minerals, like PIC, derived exclusively from DIC in surface waters, like bicarbonate. Our results demonstrate that coccolithophore PIC can incorporate DOC, previously fixed on time scales of 24 hours, which likely originated from rapidly recycled DOC as respired DIC. The AP paradigm assumes that coccolithophore PIC was formed by drawing down surface bicarbonate and “pumping” it to depth as calcium carbonate. While that indeed appears to be the dominant mechanism, we show here that DOC from just three select organic compounds (out of many that make up total DOC) appears to have been rapidly recycled into DIC and then fixed into PIC to contribute to the sinking PIC flux.

Net community production is the difference between community autotrophic primary production and total community respiration. The BCP paradigm integrates the net community production of bacterioplankton, phytoplankton, mixoplankton, and zooplankton across the microbial food web of the sea. The microbial loop cycles POC, DIC, and DOC tightly within marine plankton communities (*53*). Most of the fixed carbon originates from the DIC equilibrium species (including CO_2_, which is continually driven toward equilibrium with atmospheric CO_2_ concentrations in surface waters). Our results have demonstrated in field experiments with natural plankton communities that there can be slow rates of assimilation of three radiolabeled organics into the bulk-filtered POC fraction (roughly 0.01 to 1% of the carbon fixed via typical bicarbonate incorporation into organic matter; Table 3), consistent with previous observations of osmotrophy by many microalgal classes (see Introduction). The magnitude of the BCP is hastened by dense biominerals that become associated with sinking marine snow aggregates (*54*). Total POC (both living and dead) is ballasted by PIC calcium carbonate particles such as coccoliths (*55*) and less so by the less-dense biogenic silica (BSi) within diatom frustules (*56*). Calcium carbonate could also hasten the BCP by “protecting” POC degradation by being associated with the buffering effect of PIC particles (*57*). Osmotrophic uptake of DOC into POC and PIC coccoliths means that DOC is also being transformed into the export flux of sinking particulate carbon. Moreover, as sinking POC and PIC are remineralized at depth, the DIC released into deep waters has effectively transported surface carbon, as DOC, into the deep DIC pool via POC and PIC intermediaries. Any POC and PIC that survives the transit to the sediments is also effectively sequestering that osmotrophically fixed DOC (Fig. 6).

Regarding the AP paradigm, calcification can be attributed to multiple taxa in surface waters, such as coccolithophores, pteropods, and foraminifera. Net community calcification (NCC) refers to calcification by all calcifying organisms. In our conceptual model (Fig. 6), we are only referring to coccolithophore calcification (denoted NCC*), as influenced by osmotrophic uptake of DOC. Typically, NCC*, like NCC, draws down bicarbonate and carbonate alkalinity of surface waters and fixes that carbon into fast-sinking calcium carbonate (*58*). DOC that is osmotrophically taken up by coccolithophores in both the light and dark, then fixed into PIC, represents an additional carbon source for sinking PIC as with the BCP. The fact that it is converted from ^14^C-DOC into ^14^C-PIC in a matter of hours raises the question of what biochemical process(es) is involved. The most logical mechanism for this is that the coccolithophores themselves are assimilating the DOC [which may or may not be contributing to organic alkalinity (*59*)] and respiring some of the carbon as CO_2_, in equilibrium with ambient bicarbonate and carbonate, which is subsequently used in calcification. Another mechanism would be that other organisms (microalgae and bacteria) assimilated the DOC and respired CO_2_ into the medium whereupon the coccolithophores used it in calcification. The problem with this interpretation, however, is that any ^14^C-DOC (at initial concentrations of 10 to 100 nM in seawater) were completely respired by bacteria as ^14^C-DIC; then, the isotope dilution by unlabeled, ambient DIC (~2.2 mM) would still be on the order of 10,000× to 100,000×, which would have severely inhibited the subsequent labeling of the coccolithophore PIC on 24-hour time scales. One possible way that the labeled DOC could be quickly incorporated into PIC via bacterial respiration would be if the bacteria were epiphytic on the coccolithophores. Coccolithophore-bacteria associations have been described before (*60*, *61*), so this explanation is feasible. Nonetheless, the most likely way for DOC to be fixed quickly into PIC would be via the direct metabolism of the DOC by the coccolithophores themselves, with utilization of the respired DIC in cellular calcification. Any DOC osmotrophically fixed into sinking PIC might subsequently dissolve because of supra-lysocline dissolution, or dissolution below the lysocline (*62*). Thus, the PIC originally fixed from DOC would be released as bicarbonate at depth (another potential source of alkalinity). Similarly, if the PIC coccoliths reach the sediments, any sequestered carbon will have originated from both surface DIC and osmotrophically fixed DOC (see Fig. 6). Future research should be directed to answer how much of sinking PIC flux in the sea originates from coccolithophore osmotrophy of DOC. Specifically, it is important to identify the dominant classes of organic molecules taken up by coccolithophores in field experiments and what is the total DOC incorporation rate into PIC (as opposed to just three organic molecules as shown here). These results will help assess the relative importance of DOC osmotrophy by coccolithophores to the BCP and AP paradigms.

## MATERIALS AND METHODS

### Experimental design

The objectives of this study were to measure DOC uptake by natural assemblages of coccolithophores in NE continental shelf waters, slope waters, and oligotrophic Sargasso Sea. Underway surveying of the study region allowed overall characterization of the physical, chemical, biological, and bio-optical properties. This involved surface sampling from the ship’s nontoxic surface seawater system. At nine select stations, vertical profiles over the euphotic and sub-euphotic depths were performed of physical, biological, and chemical variables as we searched for optimum depths to sample for four time-course experiments on coccolithophore uptake of DOC compounds. Specific sampling details are described below.

### Cruise details

The work described here was performed aboard *R/V Endeavor* cruise #616, departing from, and returning to, Narragansett, RI, USA (3 to 15 July 2018). Early summer is the peak period for coccolithophore blooms in this region (*63*). The cruise track first crossed the GOM North Atlantic Time Series (GNATS) transect line (*51*). This time series was started in 1998 and has sampled physical, chemical, biological, and bio-optical properties across the GOM, between Yarmouth, Nova Scotia, Canada, and Portland, Maine, USA. From there, the cruise further sampled the GOM, then crossed Georges Bank, continental slope, and shelf waters southeast of Cape Cod, waters off of Long Island, and then a station line leading into the Sargasso Sea (Fig. 1).

### Sampling protocols

#### Underway sampling

Continuous underway measurements were made on surface seawater collected from the ship’s sea chest at 5-m depth. These data were used to characterize surface phytoplankton populations and search and identify coccolithophore-rich surface waters for subsequent depth-resolved sampling. The underway sampling methods are described elsewhere (*64*, *65*). The underway seawater was run through a shipboard: (i) debubbler (to remove optically scattering bubbles from the flow stream), (ii) SeaBird temperature/conductivity sensor (to estimate temperature and salinity), (iii) WETLabs ac-9 sensor for spectral measurements of total particulate and dissolved attenuation and absorption (at 412, 440, 480, 510, 550, 610, 630, 686, and 710 nm) [water was switched between raw streams and filtered serially through a 1-μm-pore-size filter followed by a 0.2-μm-pore-size filter to estimate the spectral absorption and attenuation of only colored dissolved organic matter (note that ac-9 was calibrated with reverse osmosis, polished water to a resistance of 18.2 megohms)], (iv) a Wyatt EOS volume-scattering sensor was used to measure the volume scattering function in the forward and backward directions at 16 angles (this stream was alternately acidified to a pH of <5.8 every few minutes to dissolve and remove the scattering of PIC, allowing us to estimate the PIC-specific backscattering of coccolithophores), (v) a WETLabs flow-through fluorometer to measure the in vivo fluorescence of chlorophyll *a*, (vi) a WETLabs flow-through fluorometer to measure fluorescent dissolved organic matter, and (vii) a HOBILabs Hydroscat II sensor for measuring spectral particulate backscattering (*b*_bp_; at 440 and 683 nm).

#### Discrete water sampling, depth profiles, and CTD/rosette casts

A total of nine stations were visited during the cruise, each of which included an optical cast using a SeaBird HyperPro hyperspectral radiometer within 2 hours of local apparent noon (*66*), secchi disc lowering, and a conductivity-temperature-depth (CTD)/rosette profile of temperature, salinity, dissolved oxygen, and beam attenuation. At eight depths, three 10-liter Niskin samples were taken for discrete measurements of chlorophyll *a* (*67*), nutrients (nitrate, nitrite, ammonium, phosphate, and silicate) (*67*), POC plus PON (*68*), PIC (*69*), BSi (*70*), and birefringence counts of coccolithophores (*68*) (the latter five analyses were done ashore, after the cruise).

#### Incubation stations

For four of the nine stations (stations 1, 3, 5, and 9), 24-hour incubation experiments (A, B, C, and D, respectively) were performed with natural populations (Fig. 1). Stations were typically occupied at about 11:00 hours EDT to optimally sample the euphotic light conditions when the sun was near peak elevation; sampling began with an optics cast and then CTD cast as above. Eight depths were sampled, and 100-ml samples were filtered onto 0.4-μm-pore-size, 25-mm-diameter polycarbonate filters for filter transfer freeze (FTF) coccolithophore counts (*71*). The FTF technique is a semiquantitative technique for microscopy and enumeration of phytoplankton onboard ship to determine rough depth profiles of phytoplankton concentration (different from the more quantitative birefringence counts described above, done ashore, after the cruise). Once the depth of maximum coccolithophore concentration was found, the Niskin bottles from that depth were drained and combined into a single, acid-cleaned polycarbonate carboy for the incubation experiments. Scanning electron microscopy was also performed after the cruise on select filtered samples for identification of coccolithophore species (*72*). Table 1 gives the location, date, depth of all the stations, and stations where incubation experiments were performed.

#### Incubation experiments with radiolabeled DOC compounds

At each station, 1 to 2 liters of ambient seawater from the combined carboy water sample above were poured into 16 intravenous flexible plastic bags [Thermo Fisher Scientific, LabTainer BioProcess Container (Waltham, MA, USA)]. Four bags each were used for incubations with each of the four radiolabeled compounds (16 bags total): ^14^C-acetate [specific activity, 1.924 MBq (μmol)^−1^; inoculum, 120 μl per bag at 1.923 mM stock concentration], ^14^C-mannitol [specific activity, 2.146 MBq (μmol)^−1^; inoculum, 120 μl per bag at 1.724 mM stock concentration], ^14^C-glycerol [specific activity, 5.92 MBq (μmol)^−1^; inoculum, 120 μl per bag at 0.625 mM stock concentration], and ^14^C-bicarbonate [specific activity, 2.146 MBq (μmol)^−1^; inoculum, 200 μl per bag at 17.24 mM stock concentration] (PerkinElmer, Waltham, MA, USA). Final concentrations of radiolabeled and ambient DOC compounds for each experiment are given in table S3.

Following inoculation, all bags were gently mixed to disperse the radiolabeled compound. The bags were placed in an incubator with light bank and day:night timer, neutral-density screening to achieve the ambient light level from the collection depth and maintained at the collection temperature with day-night cycle adjusted for the collection location and date. One of each of the four replicate bags received 5% final concentration of buffered formalin and served as a killed control. Bags were incubated for 24 hours and then brought into the darkened radioisotope van (with only dim red light). Bags were hung, and 100 to 200 ml were withdrawn and immediately filtered as “bulk samples” on a 25-mm-diameter, 0.4-μm-pore-size polycarbonate filter and subsequently processed using the microdiffusion technique (*73*, *74*) to determine the C^14^ activity in POC and PIC of the particles (see below). Next, a high-volume cell trap (HVCT; MEM-TEQ Ventures Ltd., UK) was attached to each bag, the remaining volume of 800 to 900 ml was run through the HVCT for experiments A to C, and 1800 ml was run for experiment D from the Sargasso Sea station. The concentrated particles were eluted from HVCT with ~4 ml of filtered sea water for subsequent flow cytometry (see below).

#### Sampling of ambient concentrations of specific DOC compounds

To calculate the uptake of dissolved organic compounds, the ambient concentrations of each compound (unlabeled) were measured. Niskin water samples were poured directly into acid cleaned glass scintillation vials and frozen at −20°C until analysis ashore. The concentrations of acetate, mannitol, and glycerol were determined after cruise using the methods described below. A summary of the limits of detection, accuracy, precision, and workable range of each method can be found in table S1. The equation for calculation of the uptake rates is given below.

Quantitative light microscope counts were also required for determining the concentration of coccolithophores and detached coccoliths in the field incubation sample. A volume of 50 ml was filtered onto 0.4-μm-pore-size, 25-mm-diameter polycarbonate filter and then processed according to Balch and Utgoff (*75*).

### Uptake and assimilation of DOC into POC and PIC fractions

#### Bulk phytoplankton measurements

Bulk water samples from the intravenous bags were filtered onto 25-mm-diameter, 0.4-μm-pore-size, polycarbonate, track-etched membrane filters, held in a 12-place Millipore filter tub under <5 mmHg vacuum. Following filtration, each filter was rinsed three times with filtered seawater and then given a gentle “rim rinse” following the removal of the top filter holder to remove any remaining dissolved ^14^C activity from the moist filter. Each filter was then removed for the microdiffusion protocol (*73*, *74*). Briefly, each filter was placed on the bottom of a clean scintillation vial and sealed with a rubber septum, which also held a suspended bucket containing a 25mm-diameter, glass fiber filter, saturated with 0.2 ml of phenethylamine (CO_2_ absorbent). One milliliter of 1% (by volume) phosphoric acid was injected through the rubber septum, past the suspended bucket, onto the original sample filter on the bottom of the scintillation vial to dissolve the ^14^C-PIC, converting it to ^14^C-CO_2_ gas, which diffused into the headspace. This ^14^C-CO_2_ was absorbed onto the filter in the suspended bucket over the next 24 hours as the sealed scintillation vials were gently shaken on a shaker table. After 24 hours of shaking, the vials were removed, septa were opened, and the bucket containing the glass fiber filter with absorbed ^14^C-CO_2_ (originally ^14^C-PIC) was snipped into another, clean scintillation vial, scintillation cocktail added (Ecolume, MP Biomedicals). Sample radioactivity was measured within 2 months following the cruise (see below).

#### FCM sorting of coccolithophores

A Becton Dickinson Influx Mariner FCM was used for sorting individual, naturally occurring coccolithophores in these experiments; it was housed in a specially modified sea-going shipping container. The FCM had a 488-nm coherent sapphire laser operating at 200 mW. It was configured with dual forward scatter detectors, one to collect parallel forward scattered light (PFSC), with the same polarized orientation as the laser, and one arranged orthogonally to the laser polarization to collect particle birefringence associated with cross-polarized light scatter [also known as orthogonal forward scattered light (OFSC)] (*76*). The sort region was confirmed with a culture of *Crucioplacolithus neohelis* (National Center for Marine Algae and Microbiota; CCMP298) and gated using both OFSC and relative red fluorescence (at 692 nm using a band-pass filter of ±20 nm). For a particle to be sorted, it had to be high in OFSC and contain red fluorescence (indicative of chlorophyll), and was triggered on PFSC for the particles of similar size to the cultured *C. neohelis* cells. An example of the data can be seen in fig. S6. The FCM was equipped with a 70-μm nozzle, and sorts were confirmed microscopically to be one particle per drop. Gating was optimized for particles ≳3-μm diameter. On the basis of the gating structure, the FCM sorts did not contain detached coccoliths or empty coccospheres (while the bulk samples did). For instrument operation, a NaCl solution (30 parts per thousand) was used (achieved by dissolving 210 g in 7 liters of MilliQ water, which was then 0.2 μm–filtered and buffered with NaHCO_3_ to a final pH of 8 to 8.5). Depending on the coccolithophore biomass present in the seawater sample and the amount of time available to sort them, typically 5000 to 40,000 cells were sorted and deposited into a borosilicate scintillation vial containing 1 ml of 0.2 μm–filtered seawater, which was rinsed and filtered as above for the bulk samples.

#### Sample radioactivity measurements

The radioactivity of the above filters was measured using a Tri-Carb 3110TR time-resolved liquid scintillation counter (PerkinElmer), set for transformed spectral index of external standards. The instrument was coupled to automatic efficiency correction as well as background subtraction from each of the three spectral counting regions of the counter. Static electricity associated with each vial was eliminated by the counter. True decay events from the sample were defined as being within 18-ns time difference for the two photomultiplier tubes monitoring a sample vial. The time period that the detector looks for additional pulses after the initial pulse (termed “after pulses”) was set to 75 ns. Beyond this “after-pulse” time, the scintillation events were considered to be associated with background counts. To increase the precision of the radioactivity measurements, 10 replicate counts were performed for each sample. Replicate sample counts continued until the final average count rate had an overall 95% confidence limit of ≤±0.5%. This was achieved after a total of 160,000 accumulated scintillation counts. If the accumulated counts did not reach that level in 10 min, counting of that replicate was terminated (but the count was still tabulated along with its confidence limit). This 10-replicate approach allowed us to increase the precision, reducing the SEs by a factor of 3 [= square root of (10 − 1)].

Uptake was derived from the difference between the sample average count *DPM_s_* and its associated formalin-killed blank *DPM_b_*. *Uptake* was calculated as

$Uptake=[(DPM_{s}-DPM_{b})\times 1.05]/[DPM_{tot}(V_{s}/V_{tot})T_{elap}]$ where 1.05 was the isotope discrimination factor for ^14^C compared to ^12^C; *DPM_tot_* was the radioactivity of the total counts added to the experimental sample; *V_s_* and *V_tot_* were the volumes of the experimental sample and the subsample for determination of the total activity, respectively; and *T_elap_* was the elapsed time between the moment the isotope was added until the sample was filtered. The radioactivity for the three experimental bags was then averaged within a treatment and SD calculated. Cellular uptake into PIC for bulk phytoplankton samples was calculated by dividing the uptake rates into PIC (i.e., calcification rate) from Eq. 1 by the concentration of coccolithophores in the sample. For FCM samples, the coccolithophore-specific POC and PIC production rates were calculated by taking the results of Eq. 1 and dividing them by the numbers of coccolithophores sorted by the FCM for each sample.

### Method to determine ambient acetate

An enzymatic approach was adapted to measure ambient concentrations of dissolved acetate (*27*). The assay is based on the formation of adenosine monophosphate (AMP) from adenosine triphosphate (ATP) in the presence of acetate, coenzyme A (CoA), and *S*-acetyl CoA synthetase. Frozen samples (−20°C) from the Niskin bottles (see above) were thawed at room temperature before subsampling of 1 ml into a 2-ml clean glass autosampler vial. For both samples and standards, 10 μl each of *S*-acetyl-CoA synthetase (~20 U/ml), ATP (10 mM), bovine serum albumin (200 μg liter^−1^), and CoA (10 mM) were added to the 1-ml volume. All reagents were obtained from Sigma-Aldrich Inc., St. Louis, MO, USA. The mixtures were vortexed for 5 s and then incubated on a heating block at 37°C for 1 hour. Before injection onto the high-performance liquid chromatography (HPLC) system for analysis, solutions were removed from the heating block and placed in a boiling water bath for 2 min to inactivate the *S*-acetyl CoA synthetase enzyme. For preparation of standards, a 100 μM solution of acetate in filtered, aged sea water (FASW) was prepared from 17.4 M glacial acetic acid in purified water (10 mM) adjusted to pH 8.1 with ammonium hydroxide. This was further diluted in FASW to generate standards of 0.0, 0.1, 0.5, 1.0, 5.0, 10, 15, 25, 30, and 50 μM acetate.

The instrument used for AMP quantitation was an Agilent 1200 series HPLC with G1315B diode array detector, equipped with a C18 4.6 × 250 mm, 5-μm reverse-phase column (Waters). A 0.1 M potassium phosphate (pH 6.0) mobile phase was used in isocratic mode at a flow rate of 1.0 ml min^−1^, with a column temperature of 35°C and total run time of 10 min. The injection volume was 100 μl. The diode array detector was set to 260 nm with a bandwidth of 4 nm and reference wavelength of 360 nm. A wash vial containing 0.1 M potassium phosphate (pH 6.0) was used for a syringe rinse between injections.

### Method to determine ambient mannitol

A gas chromatographic–mass spectrophotometric (GC-MS) approach, previously used to determine mannitol concentration in tissue samples (*77*), was adapted to quantify ambient mannitol in seawater. Frozen samples (−20°C) from the Niskin bottles (see above) were thawed at room temperature before subsampling of 5 ml for the analysis of mannitol. Samples and standards were lyophilized and then reconstituted in 1.5 ml of ethanol (99.5%). These samples were vortexed for 1 to 15 s, sonicated for 30 min, vortexed for another 10 to 15 s, and then sonicated for a further 30 min. Following transfer to centrifuge tubes and centrifugation for 5 min at 8000 rcf, 1.4 ml of the supernatant was removed to cleaned 2-ml glass vials. To further concentrate the samples, the ethanol solutions were dried down under a gentle nitrogen gas stream at 70°C and reconstituted in 100 μl of fresh ethanol and sonicated for 120 min. Derivatization of the mannitol was achieved by the addition of 100 μl of butylboronic acid solution (10 mg ml^−1^) part way through the sonication step. The derivatized samples were centrifuged again for 5 min at 8000 rcf before analysis. Mannitol standards were made up in 5-ml FASW with the addition of 5 μl of erythritol (120 μg ml^−1^ in methanol) as an internal standard and were processed in the same manner as the seawater samples. The same erythritol internal standard was added to each 5-ml seawater sample before lyophilization and derivatization. Mannitol, erythritol, and butylboronic acid were sourced from Sigma-Aldrich Inc., St. Louis, MO, USA.

Analysis was carried out on a Shimadzu GCMS-QP2010 GC-MS equipped with a Shimadzu SHRXI-5MS column, 30-m-long, 0.25-mm-inside-diameter, and 0.24-μm df. The GC-MS column oven temperature was programmed to start at 100°C, increased at a rate of 30°C until 200°C, then increased at a rate of 20°C to a final temperature of 300°C, and held there for 1 min. The injection temperature was 280°C, as were the ion source and interface temperatures. The injection volume was 2 μl. Helium was used for the carrier gas at a constant pressure of 7.3 psi. For quantification, the major fragment ions were mass/charge ratio (*m/z*) 127 and 253 for mannitol and *m/z* 127 for erythritol.

### Method to determine ambient free glycerol

To determine dissolved glycerol concentrations in seawater, a coupled enzymatic assay was developed by modification of existing approaches (Sigma-Aldrich FG0100) (*78*). Glycerol is phosphorylated by ATP forming glycerol 3-phosphate and adenosine diphosphate (ADP) in a reaction catalyzed by glycerokinase. Glycerol 3-phosphate is then oxidized by glycerol 3-phosphate oxidase to dihydroxyacetone phosphate and hydrogen peroxide. The basis of the glycerol quantitation was HPLC-based measurement of ATP loss and ADP production.

Glycerol (100 mM), ATP disodium salt hydrate (>99%), glycerokinase from *Cellulomonas* sp., and glycerol 3-phosphate oxidase from *Pediococcus* sp. were obtained from Sigma-Aldrich Inc., St. Louis, MO, USA. A 10.6 U ml^−1^ enzyme solution of glycerokinase was prepared by adding 0.145 mg of 44 U mg^−1^ glycerokinase to 0.604 ml of 50 mM tris-HCl (pH 7.5) solution. A 102 U ml^−1^ enzyme solution of glycerol 3-phosphate oxidase was prepared by adding 1 ml of 50 mM tris-HCl (pH 7.5) solution directly to 1.7 mg of 60 U mg^−1^ lyophilized enzyme. Two reaction mixtures were generated: reaction mix #1 (no enzyme) was prepared by adding 1 ml of 50 mM tris-HCl (pH 7.5) solution, 250 μl of 2 μM ATP, and 250 μl of 180 mM MgCl_2_ to a 4-ml vial; reaction mix #2 (with enzyme) consisted of 900 μl of 50 mM tris-HCl (pH 7.5) solution, 250 μl of 2 μM ATP, 250 μl of 180 mM MgCl_2_, 50 μl of 10.6 U ml^−1^ glycerokinase, and 50 μl of 102 U ml^−1^ glycerol 3-phosphate oxidase. Glycerol standards of 2.5 to 10 nM were prepared in FASW.

For the reaction, samples were removed from the −20°C freezer and allowed to thaw to room temperature before use. Samples, or standards, were combined in a 1:1 ratio with each reaction mixture and incubated at room temperature for 10 min before injection onto the HPLC. For the analytical sequence, 150 μl of the FASW blank was added to a 300-μl autosampler vial followed immediately by 150 μl of reaction mix #1 (no enzyme). The mixture was mixed several times by pipette aspiration. After 10 min, the blank was injected onto the HPLC. At which time, the next incubation using another 150 μl of FASW blank and 150 μl of reaction mix #2 (enzyme) occurred in a separate autosampler vial.

An Agilent 1200 series HPLC with G1315B diode array detector was used to analyze ATP and ADP concentrations at the end of the 10-min reactions. The HPLC was equipped with a C18 4.6 × 250 mm, 5-μm reverse-phase column (Waters), run with a mobile phase of 0.1 M potassium phosphate (pH 6.0) at a flow rate of 1.0 ml min^−1^ and column temperature of 35°C. The sample injection volume was 100 μl. The chromatography run time was 10 min. The diode array detector was set to 260 nm with a reference wavelength of 360 nm. A wash of 0.1 M potassium phosphate (pH 6.0) was used as a syringe rinse between samples. ATP and ADP concentrations in reaction mix #2 were corrected relative to the no enzyme control in reaction mix #1 to estimate glycerol concentrations.

### Statistical analyses and plotting software

Arithmetic means and SDs are shown in figures and tables when applicable. SD for the ratio of PIC fixation rates/mean POC fixation was calculated using standard error propagation protocols (*79*). Limits of detection, accuracy, precision, and range of analyses for measurements of acetate, mannitol, and glycerol are provided in table S1. Significant differences between sample radioactivity, after subtracting radioactivity values for killed controls, were calculated using Student’s *t* test (table S2) with an α level of 0.05 (*80*). Statistical analyses were performed using JMP statistical software (version 16; SAS Corporation, Cary, NC, USA). Plotting of oceanographic data was performed using Ocean Data View software (*81*).

## Supplementary Materials

### This PDF file includes:

Supplementary Results Figs. S1 to S6 Tables S1 to S4

## Notes

View/request a protocol for this paper from *Bio-protocol*.
