# Supplementary material for: Osmotrophy of dissolved organic compounds by coccolithophore populations: Fixation into particulate organic and inorganic carbon
Source: Sci Adv. 2023 May 24;9(21):eadf6973. doi: 10.1126/sciadv.adf6973 (PMC10208565; doi:10.1126/sciadv.adf6973)
Supplement: Supplementary file 1 — Supplementary Results Figs. S1 to S6 Tables S1 to S4 [file sciadv.adf6973_sm.pdf]

Science Advances
AAAS

Supplementary Materials for

Osmotrophy of dissolved organic compounds by coccolithophore populations: Fixation into particulate organic and inorganic carbon

William M. Balch *et al.*

Corresponding author: William M. Balch, bbalch@bigelow.org

Sci. Adv. **9**, eadf6973 (2023)
DOI: 10.1126/sciadv.adf6973

This PDF file includes:

Supplementary Results
Figs. S1 to S6
Tables S1 to S4

Supplementary Results

Additional details on environmental description of study area

Ambient environmental variables are shown as temperature-salinity plots (Figs. S1-S5). The water properties clustered into three water masses. Stations 1-3, 6 and 7 were indicative of summertime, lower-salinity, New England Shelf waters. Stations 4, 5 and 8 showed intermediate salinities and warmer temperatures, associated with New England Slope waters while station 9 T-S distributions showed warmest and saltiest conditions, representative of Sargasso Sea water (Figs. 2, S1). Concentrations of chlorophyll *a* where most elevated throughout the water column in NE Shelf waters, with clear subsurface maxima in NE Slope stations and a small subsurface maximum near the sigma-theta 26 isopycnal in the Sargasso Sea station (Fig. 2).

Profiles of five different nutrient concentrations (inorganic phosphate, silicate, nitrate, nitrite and ammonium) as a function of density are shown in Figure S1. NE Shelf waters showed measurable concentrations of phosphate and silicate throughout the water column with elevated subsurface peaks near the sigma-theta 25 isopycnal. NE Slope waters showed undetectable-to-low phosphate and silicate with pronounced subsurface peaks of inorganic phosphate and silicate between 26-27 sigma-theta isopycnals. Sargasso Sea water showed undetectable-to-low concentrations of phosphate and silicate throughout the water column except near the sigma-theta 26 isopycnal (Fig. S1). As noted above, nitrate and nitrite were barely detectable throughout most of the upper water column and showed increases at: sigma theta 25 for NE Shelf waters, sigma theta 26.5 for NE Slope waters and sigma-theta 26 for Sargasso Sea waters. Ammonium concentrations were patchily distributed, highest in the deep NE Shelf waters and close to undetectable in NE Slope and Sargasso waters (Fig. S1).

Highest extractable chlorophyll *a* concentrations were observed in the NE Shelf stations. Biogenic silica (BSi) concentrations showed highest values deep in the euphotic zone for both NE Shelf and NE Slope waters and uniformly low values at the Sargasso stations. POC, PON and PIC distributions all followed similar trends except PIC concentrations at station 3, in the center of the coccolithophore bloom, were highest of all the stations, throughout the water column (Fig. S2).

In vivo chlorophyll fluorescence showed vertical profiles (Fig. S3) similar to the extracted chlorophyll *a* profiles shown in Fig. S2, with highest values throughout the water

column in NE Shelf waters, deep chlorophyll maxima in NE Slope waters and extremely low values in Sargasso Sea waters. Beam attenuation values showed similar relative distributions as the POC distributions except for the station 3 in the coccolithophore bloom. Dissolved oxygen distributions showed most elevated concentrations in the upper layers of NE Shelf waters and generally lower concentrations at depth, particularly in the NE Slope waters at sigma-theta isopycnals between 26.5-27 (Fig. S3). Residual nitrate (= [nitrate]-[silicate]) values were close to zero or slightly negative in NE Shelf waters. In NE Slope and Sargasso waters, residual nitrate was close to zero except deep in the NE Slope waters where residual nitrate values were between 5-10μM. POC:PON molar ratios were generally highest in the surface NE Shelf and NE Slope waters (12-20), decreasing towards the Redfield molar ratio (6.62) at depth (Fig. S3F). In the Sargasso Sea profile, the POC/PON molar ratios were always elevated, ~10-25 (Fig. S3F).

Detached coccolith concentrations were uniformly low at all density surfaces except in the low-density waters of NE Shelf station 3 (bloom station). Plated coccolithophore concentrations were elevated in stations 3 and 7 in low-density surface waters with a subsurface peak in NE slope waters near the sigma-theta 26 isopycnal (Fig. S4).

Profiles of select variables are also shown as a function of the percentage of surface photosynthetically-available radiation (PAR) in order to examine their distributions across the euphotic zone (Fig. S5). The nitracline (highest gradients of nitrate concentration) consistently fell between ~1-10% of surface PAR, at similar depths as the chlorophyll maxima (Fig. S5B) except at many of the stations, there were also peaks in chlorophyll observed in shallower waters, typically above the nitracline (Fig. S5B). POC profiles as a function of percent of surface PAR (Fig. S5C) were similar to the chlorophyll profiles but with greater variability. Ambient concentrations of the acetate, mannitol and glycerol plotted as a function of light showed no consistent pattern (Fig. S5D-F).

Fig. S1.

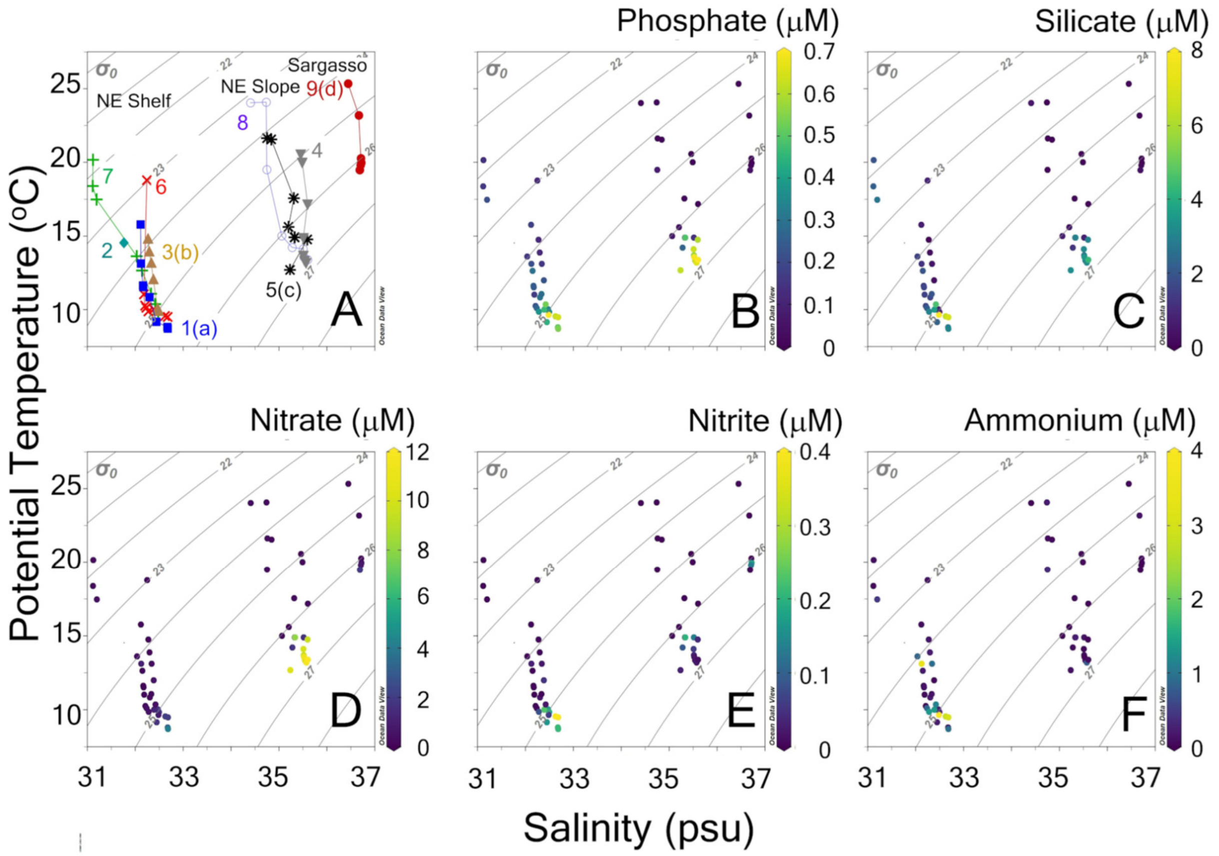

Fig. S1. Nutrient concentrations from EN616 CTD/Niskin bottle casts.
Results shown in temperature versus salinity plots for 9 stations. **(A)** Each of the station vertical profiles are grouped into New England Shelf stations, New England Slope stations and Sargasso Sea stations. Key to symbols designating each station number, shown next to respective profile: 1 (solid squares), 2 (solid diamond), 3 (solid up-pointing triangle), 4 (solid down-pointing triangle), 5 (asterisks), 6 (X symbols), 7 (+ symbols), 8 (open circles) and 9 (solid circles). Other panels are the same as panel A, but color of the symbols designates micromolar nutrient concentrations of: **(B)** inorganic phosphate, **(C)** silicate, **(D)** nitrate, **(E)** nitrite and **(F)** ammonium, as keyed to color scale to right of each panel.

Fig. S2.

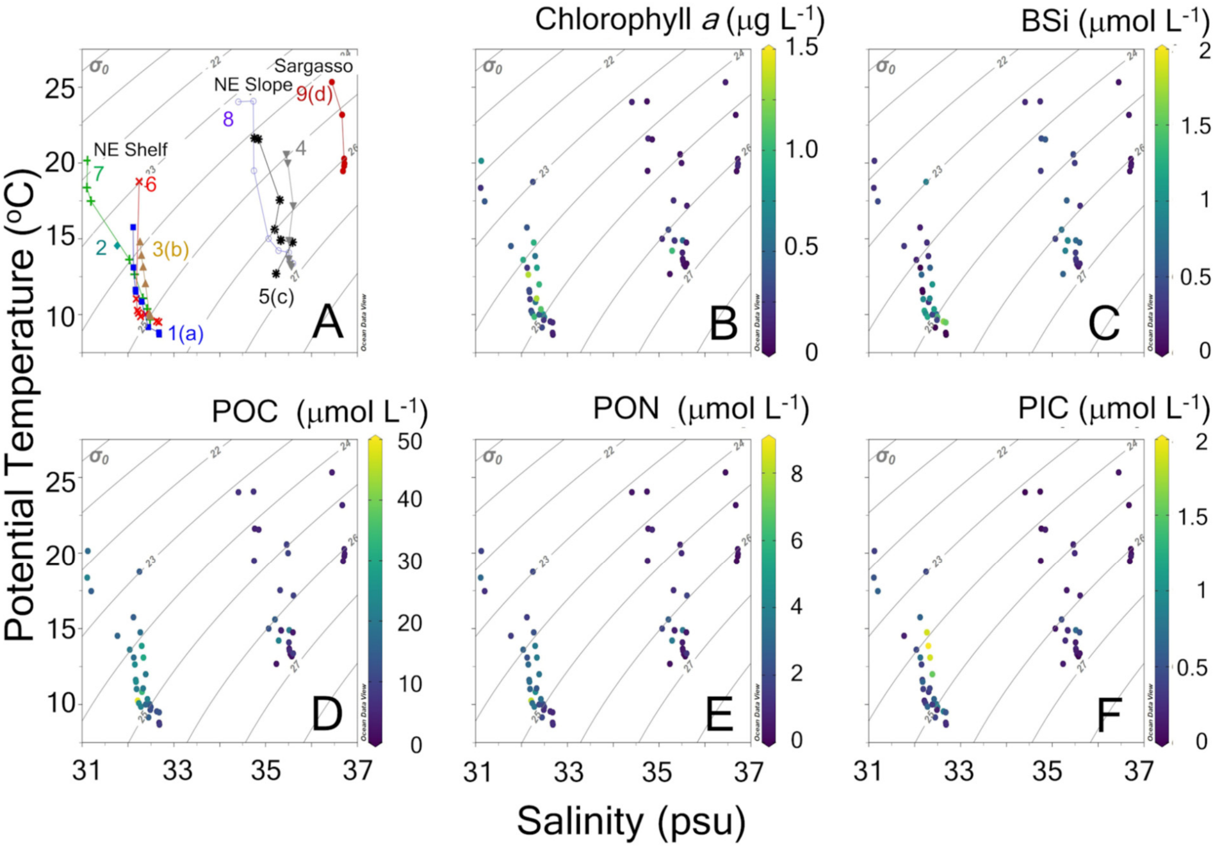

Fig. S2. Biogeochemical variables from EN616 CTD/Niskin bottle casts.
Results shown in temperature/salinity plots**.** **(A)** Temperature/salinity plots for each station of the cruise. Each of the profiles are grouped into New England Shelf stations, New England Slope stations and Sargasso Sea stations. Other panels are the same as panel A, but the Z axis shows concentrations of: **(B)** extracted chlorophyll a (µg L^{-1}), **(C)** biogenic silica (µM), **(D)** POC (µM), **(E)** PON (µM) and **(F)** PIC (µM). Symbol and color scales for plots as described in Fig. S1.

Fig. S3.

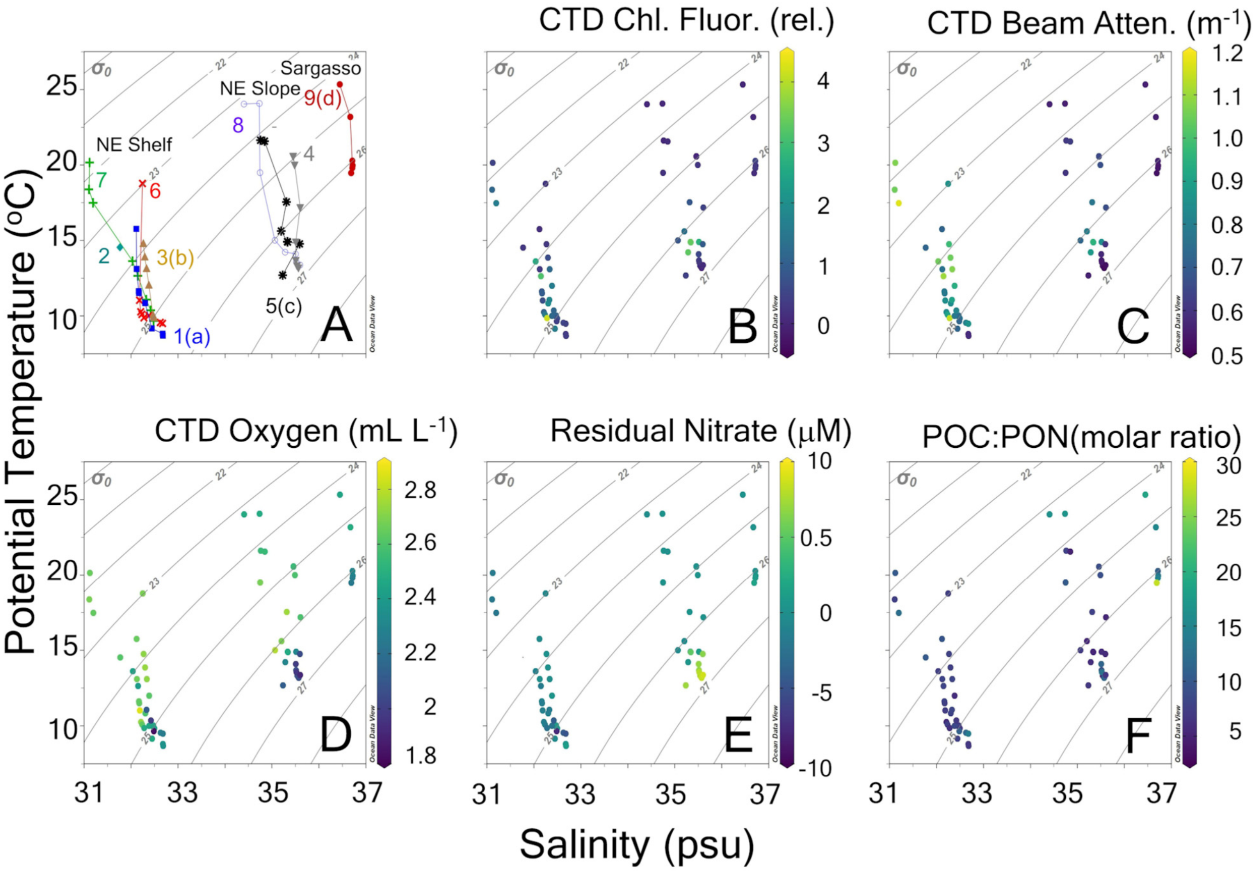

Fig. S3. CTD/Niskin bottle cast data for chlorophyll fluorescence, beam attenuation, oxygen residual nitrate and POC/PON.
Results shown in T/S plots for 9 stations taken during cruise EN616. **(A)** Each of the station vertical profiles are grouped into New England Shelf stations, New England Slope stations and Sargasso Sea stations. Other panels are the same as panel A, but the Z axis shows: **(B)** chlorophyll *a* fluorescence ($\mu g\ L^{-1}$), **(C)** beam attenuation (m^{-1}), **(D)** CTD Oxygen ($mL\ L^{-1}$), **(E)** Residual nitrate (nitrate-silicate ; μM), and **(F)** POC/PON (molar ratio). Symbol and color scales for plots as described in Fig. S1.

Fig. S4.

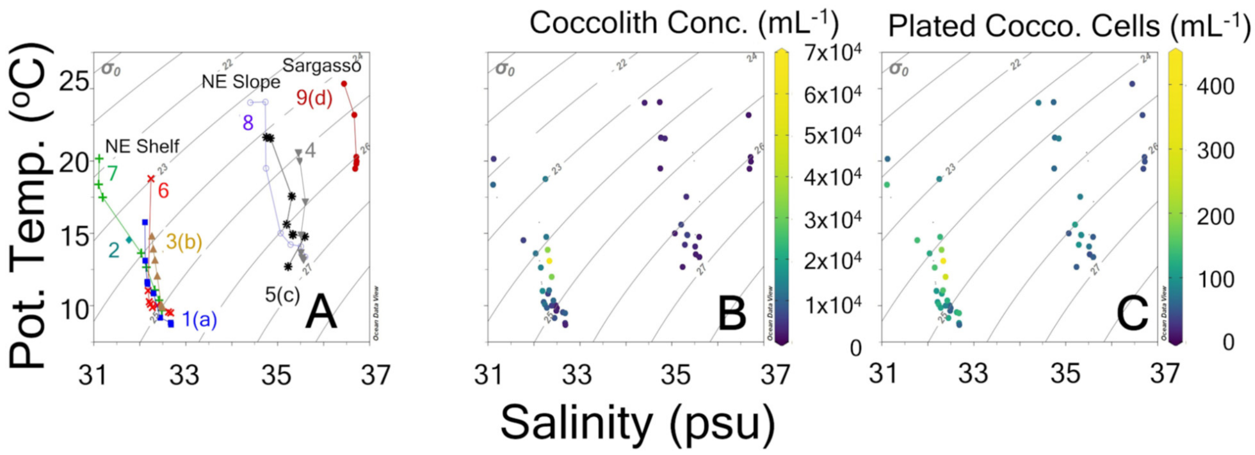

Fig S4. Coccolith and coccolithophore profiles from EN616 CTD/Niskin bottle casts. Results shown in temperature/salinity plots. **(A)** Each of the station vertical profiles are grouped into New England Shelf stations, New England Slope stations and Sargasso Sea stations. Other panels are the same as panel A, but the Z axis shows: **(B)** Coccolith concentrations (mL^{-1}) and **(C)** concentration of plated coccolithophores (mL^{-1}). Symbol and color scales for plots as described in Fig. S1.

Fig. S5.

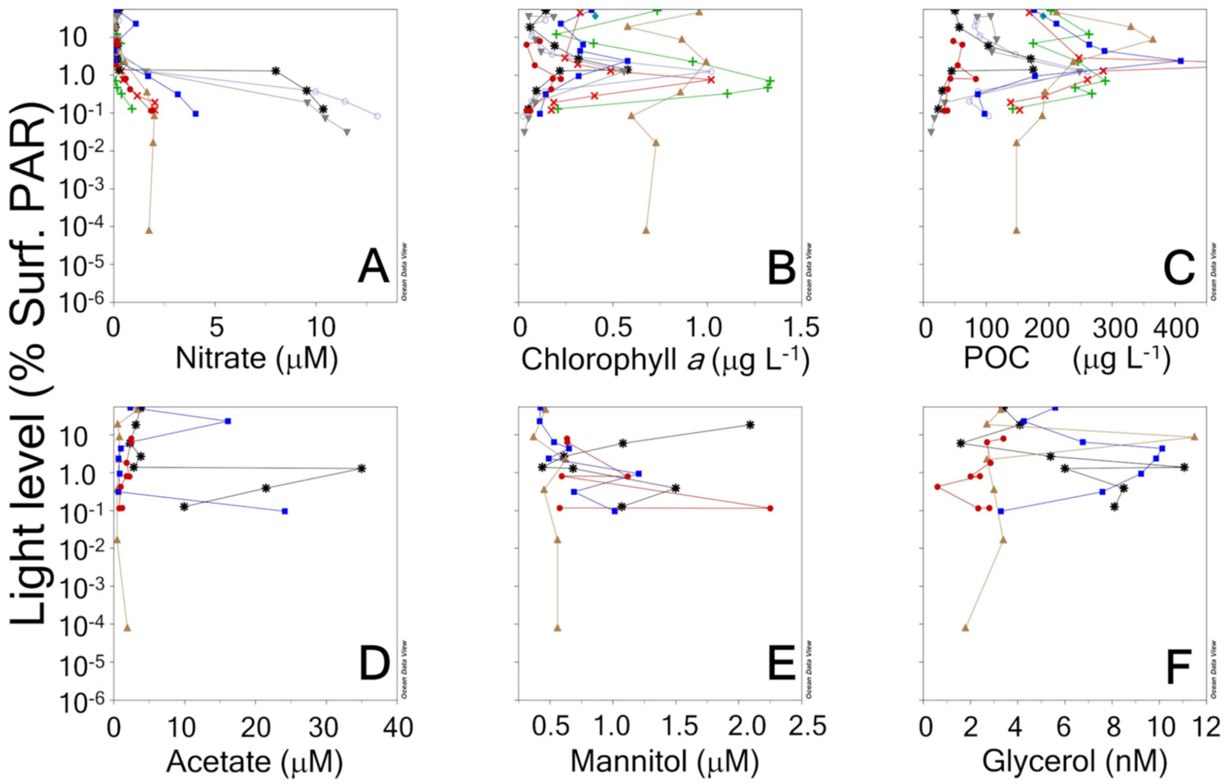

Fig. S5. Profiles of nitrate, chlorophyll, POC and DOC organics shown as a function of light from EN616 CTD/Niskin bottle casts.
Vertical profiles of % surface PAR (logarithmic scale) versus **(A)** nitrate concentration, **(B)** chlorophyll *a* concentration,**(C)** POC concentration, **(D)** acetate concentration,**(E)** mannitol concentration and **(F)** glycerol concentration. Key to symbols designating the number of each station: 1 (solid squares), 2 (solid diamond), 3 (solid up-pointing triangle), 4 (solid down-pointing triangle), 5 (asterisks), 6 (X symbols), 7 (+ symbols), 8 (open circles) and 9 (solid circles).

Fig. S6.

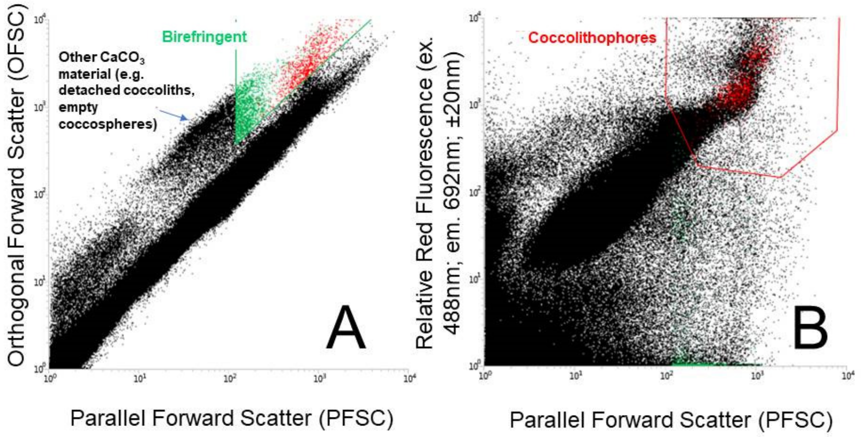

Fig. S6. Flow cytometric dot plots from Station 1 (Gulf of Maine 6 July, 2018).
A) OFSC vs PFSC and **B)** PFSC vs red fluorescence. The sort gates targeted coccolithophore red fluorescence (red dots; indicative of chlorophyll *a*) and birefringence (green dots – suggestive of $CaCO_3$ composition). FCM gates were set using a laboratory culture of the coccolithophore, *Crucioplacolithus neohelis* that was brought along on the cruise.

Table S1.

Compound	Limit of detection (μM)	Accuracy (μM)	Precision (μM)	Range (μM)
Acetate	0.039	0.091	0.235	0.039 - 30
Mannitol	0.033	0.0025	0.05	0.033 - 64
Glycerol	0.0004	0.0002	0.0009	0.0004 - 0.010

Table S1.
Summary of statistics for the methods for measuring dissolved organic carbon compounds. This table illustrates the limits of detection, accuracy, precision and workable range of methods to measure ambient acetate, mannitol and glycerol in seawater. These results are based on replicate measurements made throughout the study.

Table S2.

						POC							PIC						
Exp	Stn	Date	Location	Depth (m)	Compound	Avg Killed Control (DPM)	Killed Control Std Dev (DPM)	Avg Samp. (DPM)	Samp. Std Dev (DPM)	**Sample-Killed Control (DPM)**	Std Dev Diff (Err. Prop.)	**SNR**	Avg Killed Control (DPM)	Killed Control Std. Dev. (DPM)	Avg Samp. (DPM)	Samp. Std Dev (DPM)	**Sample-Killed Control (DPM)**	Std Dev Diff (Err. Prop.)	**SNR**
**Bulk samples**																			
a	1	7/5/2018	43° 43.1'N 067° 12.4'W	26	^{14}C-Acetate	1553.4	13.4	126814.3	285.5	**125260.9**	285.8	438.24	79.8	4.0	325.3	7.0	**245.5**	8.1	30.30
					^{14}C Mannitol	281.1	4.5	145371.0	348.9	**145089.9**	348.9	415.80	87.1	2.9	231.4	6.1	**144.3**	6.7	21.46
					^{14}C-Glycerol	1555.7	10.1	108366.5	279.2	**106810.8**	279.4	382.34	102.9	4.5	200.6	6.5	**97.7**	7.9	12.31
					^{14}C-HCO3$^-$	95.9	3.7	264134.8	1129.6	**264038.9**	1129.6	233.74	2357.1	18.3	6795.4	22.1	**4438.3**	28.6	154.95
b	3	7/7/2018	41° 41.1'N 066° 31.1'W	12	^{14}C-Acetate	7128.7	33.0	240107.7	731.6	**232979.0**	732.4	318.11	155.3	4.3	396.1	5.8	**240.8**	7.3	33.17
					^{14}C Mannitol	4476.9	15.1	204601.3	479.6	**200124.4**	479.8	417.06	134.6	4.4	347.6	7.5	**213.0**	8.7	24.46
					^{14}C-Glycerol	744.6	9.7	59212.6	138.1	**58468.0**	138.5	422.30	126.6	3.1	216.7	5.4	**90.1**	6.2	14.44
					^{14}C-HCO3$^-$	1242.1	8.5	217041.7	346.5	**215799.6**	346.6	622.70	5801.3	33.5	23266.3	57.8	**17465.0**	66.8	261.56
c	5	7/9/2018	39° 41.77'N 070° 54.0'W	41	^{14}C-Acetate	256.9	4.7	161746.9	514.3	**161490.0**	514.4	313.96	67.2	2.5	427.3	8.1	**360.1**	8.5	42.53
					^{14}C Mannitol	318.6	5.5	192001.1	527.2	**191682.5**	527.2	363.58	63.7	2.3	301.4	6.4	**237.7**	6.8	34.89
					^{14}C-Glycerol	941.5	7.0	148478.7	280.6	**147537.2**	280.7	525.67	104.5	3.4	210.9	6.2	**106.4**	7.0	15.14
					^{14}C-HCO3$^-$	94.4	3.6	64919.3	131.3	**64824.9**	131.4	493.49	2114.9	21.7	4530.0	23.0	**2415.1**	31.6	76.47
d	9	7/13/2018	36° 59.4'N 067° 59.3'W	131	^{14}C-Acetate	360.0	4.9	13834.2	33.2	**13474.2**	33.6	401.30	16.6	2.2	65.6	3.7	**49.0**	4.3	11.42
					^{14}C Mannitol	190.5	3.6	8351.0	33.1	**8160.5**	33.3	245.33	23.0	2.6	34.0	3.6	*11.0*	4.4	2.49
					^{14}C-Glycerol	323.0	5.8	16127.0	44.9	**15804.0**	45.3	349.14	14.9	1.3	30.9	2.7	**16.0**	3.0	5.32
					^{14}C-HCO3$^-$	487.4	10.4	530.8	12.9	*43.4*	16.6	2.62	3216.6	24.0	3667.9	12.9	**451.3**	27.3	16.56
**FCM-Sorted Samples**																			
a		7/5/2018	43° 43.1'N 067° 12.4'W	26	^{14}C-Acetate	139.1	4.9	66.6	5.8	-72.5	7.6	-9.53	76.6	2.7	84.1	4.1	7.5	4.9	1.51
					^{14}C Mannitol	113.8	4.3	128.2	6.4	14.4	7.8	1.86	104.0	1.6	107.2	4.1	3.2	4.4	0.74
					^{14}C-Glycerol	71.0	3.9	78.9	4.6	7.9	6.0	1.32	123.5	3.4	141.0	5.3	*17.5*	6.3	2.77
					^{14}C-HCO3$^-$	69.7	3.4	1929.4	13.8	**1859.7**	14.3	130.36	312.2	4.8	1322.2	10.5	**1010.0**	11.6	87.35
b		7/7/2018	41° 41.1'N 066° 31.1'W	12	^{14}C-Acetate	72.6	2.5	130.4	4.7	**57.8**	5.3	10.89	149.0	3.4	156.1	5.9	7.1	6.9	1.04
					^{14}C Mannitol	75.7	2.8	109.9	4.9	**34.2**	5.6	6.06	172.1	5.4	140.4	6.9	-31.7	8.8	-3.61
					^{14}C-Glycerol	53.7	1.6	75.6	3.6	**21.9**	4.0	5.52	190.2	5.4	130.7	7.2	-59.5	9.0	-6.61
					^{14}C-HCO3$^-$	62.7	2.6	2878.5	16.7	**2815.8**	16.9	166.32	179.8	5.5	2923.9	18.1	**2744.1**	18.9	145.00
c		7/9/2018	39° 41.77'N 070° 54.0'W	41	^{14}C-Acetate	63.4	2.8	94.4	4.4	**31.0**	5.2	5.96	65.2	2.0	87.0	3.5	**21.8**	4.1	5.34
					^{14}C Mannitol	195.3	5.8	615.6	9.9	**420.3**	11.5	36.61	69.1	2.0	80.8	4.0	*11.7*	4.5	2.59
					^{14}C-Glycerol	72.8	2.9	222.3	5.3	**149.5**	6.0	24.89	28.0	2.4	29.2	3.4	1.2	4.1	0.28
					^{14}C-HCO3$^-$	212.4	6.6	695.3	9.9	**482.9**	11.9	40.60	78.0	2.9	108.7	4.1	**30.7**	5.0	6.11
d		7/13/2018	36° 59.4'N 067° 59.3'W	131	^{14}C-Acetate	42.3	2.6	73.7	3.5	**31.4**	4.4	7.22	14.6	1.4	15.9	2.2	1.3	2.6	0.49
					^{14}C Mannitol	43.6	2.0	74.9	3.3	**31.3**	3.9	8.12	20.9	3.4	17.8	4.0	-3.1	5.2	-0.60
					^{14}C-Glycerol	34.9	1.4	102.9	4.1	**68.0**	4.4	15.58	26.1	1.9	15.1	2.9	-11.0	3.5	-3.17
					^{14}C-HCO3$^-$	61.9	3.8	70.9	4.9	9.0	6.1	1.46	32.6	2.0	31.0	3.0	-1.6	3.5	-0.44

Key to differences in radioactivity of experimental count from killed control

- >10,000 DPM
- 1,000 to 10,000 DPM
- 100 to 1,000 DPM
- 10 to 100 DPM
- up to 10 DPM
- No difference

	POC CV Killed control (%)	POC CV sample (%)	PIC CV Killed control (%)	PIC CV sample (%)
**Bulk samples**	1.570	0.401	4.260	2.967
**Sorted samples**	4.190	3.991	4.897	7.326
**Total Bulk & Sorted**	2.880	2.196	4.578	5.146
**Grand Total (all samples, killed controls, experimental, PIC and POC)**				3.700

Table S2. Radioactivity signals in experiments.

Relative uptake of ^{14}C organics (and bicarbonate) based on radioactivity of samples (DPM's) following 24h incubations plus difference between average of triplicate samples minus formalin-killed controls. Standard deviations are shown for 10-replicate counts of each sample. Standard error of the difference between the experimental and killed-control DPMs is calculated using standard error propagation calculations (79). Signal-to-noise ratio (SNR) of the isotopic measurement is estimated as the difference in counts between experimental and killed control samples, normalized by the error-propagated standard deviation. Statistically significant sample radioactivity with values greater than the killed control are designated in color, keyed to the range of DPMs given at bottom of the table. For colored cells in bold-face font, the significance is based on Student's T test for comparison of two means (experimental and blank treatments; critical $t_{0.05\,(1),2} = 2.920$; $P<0.05$). For colored cells with italic font, the significance is based on Student's T test for comparison of two means (experimental and blank treatments; critical $t_{0.10\,(1),2} = 1.886$; $P<0.10$). Average coefficients of variation (%) are tallied at bottom for bulk samples, sorted samples or all samples.

Table S3.

Exp. number	A				B				C				D			
DOC Compound	^{14}C-labeled (μM)	unlabeled (μM)	Total (μM)	Frac. Labeled	^{14}C-labeled (μM)	unlabeled (μM)	Total (μM)	Frac. Labeled	^{14}C-labeled (μM)	unlabeled (μM)	Total (μM)	Frac. Labeled	^{14}C-labeled (μM)	unlabeled (μM)	Total (μM)	Frac. Labeled
**Acetate**	0.231	0.676	0.907	**0.255**	0.231	0.813	1.040	**0.221**	0.231	3.820	4.050	**0.057**	0.231	0.782	1.010	**0.228**
**Mannitol**	0.207	0.492	0.699	**0.296**	0.207	0.370	0.577	**0.359**	0.207	0.612	0.819	**0.253**	0.207	2.250	2.460	**0.084**
**Glycerol**	7.50E-02	9.87E-03	8.49E-02	**0.884**	7.50E-02	0.012	0.087	**0.867**	7.50E-02	5.40E-03	0.080	**0.933**	0.078	0.002	0.081	**0.971**
**Bicarbonate**	1.410	2029.536	2030.946	**6.94E-04**	3.45	2029.54	2032.98	**1.70E-03**	3.450	2.22E+03	2.22E+03	**1.56E-03**	3.015	2.31E+03	2.31E+03	**1.30E-03**

Table S3. DOC concentrations and labeled fraction in each experiment.
Concentration of labeled, unlabeled and total DOC compounds present in each experiment. Experiments A-D are arranged in columns. Also shown are the fractions of each compound that were ^{14}C -labeled.

Table S4.

	DOC compound	Indep. Variable	Slope	SE slope	Intercept	intercept	RMSE	R^2	F ratio	P
**A.**	**Acetate**	Beam atten. (m^{-1})	-2.88E-14	1.66E-14	3.14E-14	1.37E-14	6.49E-15	0.599	2.99	0.226
	**Mannitol**	Beam atten. (m^{-1})	-7.46E-15	1.51E-15	7.86E-15	1.25E-15	5.89E-16	0.924	24.45	***0.039***
	**Glycerol**	Beam atten. (m^{-1})	-3.38E-16	4.89E-17	3.48E-16	4.04E-17	1.91E-17	0.960	47.91	***0.020***
	**Bicarbonate**	Beam atten. (m^{-1})	-5.39E-11	3.17E-11	5.60E-11	2.62E-11	1.24E-11	0.590	2.89	0.232
**B.**	**Acetate**	Sample depth (m)	1.10E-16	7.82E-17	2.49E-15	5.48E-15	7.27E-15	0.498	1.98	0.294
	**Mannitol**	Sample depth (m)	3.23E-17	2.94E-18	1.72E-16	2.06E-16	2.73E-16	0.984	120.70	***0.008***
	**Glycerol**	Sample depth (m)	1.47E-18	1.82E-19	7.70E-19	1.27E-17	1.69E-17	0.969	61.90	***0.016***
	**Bicarbonate**	Sample depth (m)	2.75E-13	7.42E-14	-1.74E-12	5.20E-12	6.90E-12	0.873	13.77	0.066

Table S4. Statistics for linear, least squares regression and F tests for the relationship between sample turbidity or sample depth versus bulk DOC incorporation into PIC. (A) Beam attenuation (m^{-1}; independent variable) versus bulk incorporation of DOC compound/bicarbonate into PIC (mol $cell^{-1}$ d^{-1}; dependent variable) and (B) sample depth (m; independent variable) versus bulk incorporation of DOC compound/bicarbonate into PIC (mol $cell^{-1}$ d^{-1}; dependent variable). For all experiments, the sample size was 4. Statistically significant relationships are designated in italicized, bold font for P values <0.05.
